# DiLizium: A Two-Party Lattice-Based Signature Scheme

**DOI:** 10.3390/e23080989

**Published:** 2021-07-30

**Authors:** Jelizaveta Vakarjuk, Nikita Snetkov, Jan Willemson

**Affiliations:** 1 Cybernetica AS, Mäealuse 2/1, 12618 Tallinn, Estonia; nsnetkov@cyber.ee (N.S.); janwil@cyber.ee (J.W.); 2 STACC OÜ, Narva mnt 20, 51009 Tartu, Estonia

**Keywords:** digital signatures, distributed signing, threshold signatures, lattice-based cryptography, Fiat–Shamir with aborts, post-quantum cryptography

## Abstract

In this paper, we propose DiLizium: a new lattice-based two-party signature scheme. Our scheme is constructed from a variant of the Crystals-Dilithium post-quantum signature scheme. This allows for more efficient two-party implementation compared with the original but still derives its post-quantum security directly from the Module Learning With Errors and Module Short Integer Solution problems. We discuss our design rationale, describe the protocol in full detail, and provide performance estimates and a comparison with previous schemes. We also provide a security proof for the two-party signature computation protocol against a classical adversary. Extending this proof to a quantum adversary is subject to future studies. However, our scheme is secure against a quantum attacker who has access to just the public key and not the two-party signature creation protocol.

## 1. Introduction

Ever since Peter Shor proposed an algorithm that was able to efficiently break most of the classical asymmetric cryptographic primitives such as RSA or ECDSA in the 1990s [1,2], research has been conducted to find quantum-resistant replacements. This work has recently been coordinated by the U.S. National Institute of Standards and Technology (NIST). In 2016, NIST announced an effort to standardise some of the proposed public key encryption algorithms, key-establishment algorithms, and signature schemes [3].

The primary focus of NIST is to obtain drop-in replacements for the current standardised primitives in order to ease the transition. However, not all of the current application areas are covered by the standardisation process.

One important class of examples is threshold signatures. In a (t,n)-threshold scheme, the secret key is shared between *n* users/devices. To create a valid signature, a subset of *t* users/devices should collaborate and use their secret key shares. Over the years, threshold versions of a number of major cryptographic algorithms including RSA and (EC)DSA have been studied [4,5,6,7,8]. Recent interest in threshold versions of ECDSA has been influenced by applications in blockchains. However, our motivation stems more from server-assisted RSA signatures, as proposed in the scheme by Buldas et al. [9]. This scheme has been implemented in a Smart-ID mobile application and was recognised as a qualified signature creation device (QSCD) in November 2018 [10]. In 2021, the number of Smart-ID users in the Baltic countries was estimated at 2.9 million [11].

In 2019, D. Cozzo and N. Smart analysed a number of NIST post-quantum standardisation candidates and concluded that none of them provide a threshold implementation that would be comparable in efficiency to threshold RSA or even ECDSA [12].

The goal of this research is to find a suitable version of one of the NIST signature candidates (Crystals-Dilithium) that would allow for a more efficient two-party implementation but would still provide post-quantum security. From this, we propose a concrete/specific two-party signature scheme and prove its security in this paper.

To be able to compute the private key or a signature forgery having access only to the public key, the attacker would need to solve Module Learning With Errors, Rejected Module Learning With Errors, and Module Short Integer Solution problems. These are considered hard even for quantum computers. We present security proof for the two-party signing protocol itself; however, it only provides security against a classical attacker. Extending the proof to cover quantum attackers is a task for future work.

### Contributions

In this paper, we construct DiLizium, a lattice-based two-party signature scheme that follows the Fiat–Shamir with Aborts (FSwA) paradigm, and prove the security of the proposed scheme in the classical random oracle model. The security of the proposed signature scheme relies on the hardness of solving the Module-LWE and Module-SIS problems. Our two-party signature protocol is based on the scheme described in the paper by Kiltz et al., Appendix B [13]. Initially, we attempted to construct a two-party version of the Crystals-Dilithium digital signature submitted to the NIST PQC completion. However, we concluded that there are no straightforward approaches for modification thereof to the distributed version. Solutions require using a two-party computation protocol, which increases not only the signing time but also the communication complexity. The simplified version of the Crystals-Dilithium scheme from [13] is easier to work with because there are no additional bit decomposition algorithms used. Having a bit decomposition protocol would require an additional secure two-party computation protocol that would allow client and server to jointly compute high-order bits without revealing their private intermediate values. That would lead to an increased number of communication rounds and usage of additional security assumptions.

Additionally, DiLizium scheme does not require sampling from a discrete Gaussian distribution. We decided to use the scheme with uniform distribution because the Gaussian distribution is known to be hard to implement securely [14,15]. Our work follows the approach from [16], but instead of using homomorphic commitments, we decided to use the homomorphic hash function. We wanted to find an alternative to the commitment scheme introduced in [16] to increase the computational efficiency of the signature scheme. Due to the way our scheme is constructed, in the security proof, we rely on rejected Module-LWE, which is a non-standard security assumption, and it is not used in this work [16].

## 2. Related Work

In 2020, NIST announced fifteen third-round candidates for the PQC competition, of which seven were selected as finalists and eight were selected as alternate candidates [17]. The finalists in the digital signature category were Crystals-Dilithium [18], Falcon [19], and Rainbow [20]. Crystals-Dilithium and Falcon are both lattice-based signature schemes. Falcon has better performance, signature, and key sizes; however, its implementation is more complex, as it requires sampling from the Gaussian distribution and it utilises floating-point numbers to implement an optimised polynomial multiplication [19]. The performance of a Crystals-Dilithium signature scheme is slightly slower, and the signature and key sizes are larger than the ones in Falcon; however, the signature scheme itself has a simpler structure [18]. Rainbow is a multivariate signature scheme with fast signing and verifying processes. The size of the Rainbow signature is the shortest among the finalists; however, the public key size is the largest [20].

Due to the interest aroused by the PQC competition, several works were proposed that introduce lattice-based threshold signatures and lattice-based multisignatures. The works [21,22,23,24,25,26] focused on creating multisignatures that followed the FSwA paradigm. These schemes use rejection sampling, due to which the signing process is repeated until a valid signature is created. Additionally, intermediate values produced during the signature generation process need to be kept secret until the rejection sampling has been completed. There are currently no known techniques to prove the security of FSwA signatures if intermediate values are published before the rejection sampling is performed [16]. In multisignatures [21,22,23,24,25], intermediate values are published before the rejection sampling is completed, which leads to incomplete security proofs in these works [16]. The work by M. Fukumitsu and S. Hasegawa [26] solves the problem with aborted executions of the protocol by introducing a non-standard hardness assumption (rejected Module-LWE).

In 2019, D. Cozzo and N. Smart analysed the second round NIST PQC competition signature schemes to determine whether it is possible to create threshold versions of these signature schemes [12]. The authors proposed a possible threshold version for each of the schemes using only generic Multiparty Computation (MPC) techniques, such as linear secret sharing and garbled circuits. As a result, the authors proposed that the most suitable signature scheme is Rainbow, which belongs to the multivariate family. The authors described a threshold version of Crystals-Dilithium, which is estimated to take around 12 s to produce a single signature. The authors explained that the problems with performance arise from the fact that the signature scheme consists of both linear and nonlinear operations and that it is inefficient to switch between these representations using generic MPC techniques. However, the goal of the current work is to focus on the two-party scenario. This means that some of the difficulties in D. Cozzo and N. Smart paper can be avoided.

R. Bendlin, S. Krehbiel, and C. Peikert proposed threshold protocols for generating a hard lattice with trapdoor and sampling from the discrete Gaussian distribution using the trapdoor [27]. These two protocols are the main building blocks for the Gentry–Peikert–Vaikuntanathan (GPV) signature scheme (based on hash-and-sign paradigm), where generating a hard lattice is needed for the key generation and Gaussian sampling is needed for the signing process. M. Kansal and R. Dutta proposed a lattice-based multisignature scheme with a single round signature generation that has key aggregation and signature compression in [28]. The underlying signature scheme follows neither the hash-and-sign nor FSwA paradigms, which are the main techniques used to construct lattice-based signature schemes.

In 2020, I. Damgård, C. Orlandi, A. Takahashi, and M. Tibouchi proposed a lattice-based multisignature and distributed signing protocols that are based on the Dilithium-G signature scheme [16]. Dilithium-G is a version of the Crystals-Dilithium signature that requires sampling from a discrete Gaussian distribution [29]. The work contains complete classical security proofs for the proposed schemes. The work solves the problem with the aborted executions by using commitments such that, in the case of an abort, only commitment is published, the intermediate value itself stays secret. The proposed distributed signature scheme could potentially fit the Smart-ID framework; however, some questions need to be addressed. More precisely, the scheme is based on a modified version of Crystals-Dilithium from the NIST PQC competition project and uses Gaussian sampling. It is known that generating samples from the Gaussian distribution is nontrivial, which means that the insecure implementation may lead to side-channel attacks [30]. The open question is whether it is possible to use a version of the scheme more similar to the one being submitted to the NIST PQC competition.

## 3. Preliminaries

### 3.1. Notation

- Let Z be a ring of all integers. $Z_{q}=Z/qZ$ denotes a ring of residue classes modulo *q*. Z[x] denotes a ring of polynomials in the variable *x* with integer coefficients.
- *R* denotes a quotient ring $Z\left[x\right]/\left(x^{n}+1\right)$, where n∈N and $R_{q}$ denotes a quotient ring $Z_{q}\left[x\right]/\left(x^{n}+1\right)$, where n∈N.
- Polynomials are denoted in italic lowercase *p*. $p\in R_{q}$ is a polynomial of degree bound by *n*: $p=p_{0}+p_{1}x+\ldots +p_{n-1}x^{n-1}$. It can also be expressed in a vector notation through its coefficients ($p_{0},p_{1},\ldots ,p_{n-1}$).
- Vectors are denoted in bold lowercase v. $v\in R_{q}^{n}$ is a vector of dimension *n*: $v=(v_{0},\ldots ,v_{n-1})$, where each element $v_{i}$ is a polynomial in $R_{q}$.
- Matrices are denoted in bold uppercase A. $A\in R_{q}^{n\times m}$ is a n×m matrix with elements in $R_{q}$.
- For an even positive integer α and for every x∈Z, define $x^{\prime }=x\;\mathrm{mod}{\;}^{\pm }\alpha$, as $x^{\prime }$ in the range $-\frac{\alpha }{2}<x^{\prime }\leq \frac{\alpha }{2}$ such that $x^{\prime }\equiv x\;\left(\mathrm{mod}\;\alpha \right)$. For an odd positive integer α and for every x∈Z, define $x^{\prime }=x\;\mathrm{mod}{\;}^{\pm }\alpha$, as $x^{\prime }$ in the range $-\frac{\alpha -1}{2}\leq x^{\prime }\leq \frac{\alpha -1}{2}$ such that $x^{\prime }\equiv x\;\left(\mathrm{mod}\;\alpha \right)$. For any positive integer α, define $x^{\prime }=x\;\mathrm{mod}\;\alpha$, as $x^{\prime }$ in the range $0\leq x^{\prime }<\alpha$ such that $x^{\prime }\equiv x\;\left(\mathrm{mod}\;\alpha \right)$.
- For an element $p=p_{0}+p_{1}x+\ldots +p_{n-1}x^{n-1}\in R_{q}$, its $l_{2}$ norm is defined as ${\left||p|\right|}_{2}=(\sum_{i}|p_{i}{{|}^{2})}^{\frac{1}{2}}$.
- For an element $x\in Z_{q}$, its infinity norm is defined as ${\left||x|\right|}_{\infty }=\left|x\;\mathrm{mod}{\;}^{\pm }q\right|$, where |x| denotes the absolute value of the element. For an element $p=p_{0}+p_{1}x+\ldots +p_{n-1}x^{n-1}\in R_{q}$, ${\left||p|\right|}_{\infty }=\max_{i}\left|\right|p_{i}{\left|\right|}_{\infty }$. Similarly for an element $v=\left(p_{0},\ldots ,p_{n}\right)\in R_{q}^{n}$, ${\left||v|\right|}_{\infty }=\max_{i}\left|\right|p_{i}{\left|\right|}_{\infty }$.
- $S_{\eta }$ denotes a set of all elements p∈R such that ${\left||p|\right|}_{\infty }\leq \eta$.
- a←A denotes sampling an element uniformly at random from the set *A*.
- a←χ(A) denotes sampling an element from the distribution χ defined over the set *A*.
- ⌈x⌉ denotes mapping *x* to the least integer greater than or equal to *x* (e.g., ⌈5.2⌉=6).
- The symbol ⊥ is used to indicate a failure or rejection.

### 3.2. Definitions of Lattice Problems

**Definition** **1(Decisional Module-LWE (**q,n,m,η,χ**)).** *Let χ be an error distribution, given a pair $\left(A,t\right)\in \left(R_{q}^{n\times m}\times R_{q}^{n}\right)$ decide whether it was generated uniformly at random from $R_{q}^{n\times m}\times R_{q}^{n}$ or it was generated as $A\leftarrow R_{q}^{n\times m}$, ($s_{1},s_{2})\leftarrow \chi \left(S_{\eta }^{m}\times S_{\eta }^{n}\right)$ and $t:={\mathrm{As}}_{1}+s_{2}$.*

The advantage of adversary A in breaking decisional Module-LWE for the set of parameters (q,n,m,η,χ) can be defined as follows:

$$\begin{array}{c} {\mathrm{Adv}}_{\left(q,n,m,\eta ,\chi \right)}^{\mathrm{Dec}-\mathrm{MLWE}}\left(A\right):=|\mathrm{Pr}\left[b=1:A\leftarrow R_{q}^{n\times m},\left(s_{1},s_{2}\right)\leftarrow \chi \left(S_{\eta }^{m}\times S_{\eta }^{n}\right),t:={\mathrm{As}}_{1}+s_{2},b\leftarrow A\left(A,t\right)\right]\\ -\mathrm{Pr}\left[b=1:A\leftarrow R_{q}^{n\times m},t\leftarrow R_{q}^{n},b\leftarrow A\left(A,t\right)\right]|. \end{array}$$

**Definition** **2(Computational Module-LWE** **(**q,n,m,η,χ**)).** *Let χ be an error distribution, given a pair $\left(A,t\right)\in \left(R_{q}^{n\times m}\times R_{q}^{n}\right)$, where $A\leftarrow R_{q}^{n\times m}$, ($s_{1},s_{2})\leftarrow \chi \left(S_{\eta }^{m}\times S_{\eta }^{n}\right)$, and $t:={\mathrm{As}}_{1}+s_{2}$ when finding a vector $s_{1}$.*

The advantage of adversary A in breaking computational Module-LWE for the set of parameters (q,n,m,η,χ) can be defined as follows:

$${\mathrm{Adv}}_{\left(q,n,m,\eta ,\chi \right)}^{\mathrm{Com}-\mathrm{MLWE}}\left(A\right):=\mathrm{Pr}[s_{1}=s_{1}^{\prime }:A\leftarrow R_{q}^{n\times m},\left(s_{1},s_{2}\right)\leftarrow \chi \left(S_{\eta }^{m}\times S_{\eta }^{n}\right),t:={\mathrm{As}}_{1}+s_{2},s_{1}^{\prime }\leftarrow A\left(A,t\right)].$$

**Definition** **3(Module-SIS** **(**q,n,m,η**)).** *Given a uniformly random matrix*$A\leftarrow R_{q}^{n\times m}$, *find a vector*$x\leftarrow R_{q}^{n+m}$*such that*$[\begin{array}{c} A|I \end{array}]\cdot x=0$*and*${0<||x||}_{\infty }\leq \eta$.

The advantage of adversary A in breaking Module-SIS for the set of parameters (q,n,m,η) can be defined as follows:

$${\mathrm{Adv}}_{\left(q,n,m,\eta \right)}^{\mathrm{MSIS}}\left(A\right):=\mathrm{Pr}[ [\begin{array}{c} A|I \end{array}]\cdot x=0\;\mathrm{and}\;{0<||x||}_{\infty }\leq \eta :A\leftarrow R_{q}^{n\times m},x\leftarrow A\left(A\right)].$$

Additionally, we define the rejected Module-LWE assumption adapted from [26].

**Definition** **4(Rejected Module-LWE**  **(**q,n,m,γ,χ,β**)).** *Let χ be an error distribution, and let C be a set of all challenges. Let $A\leftarrow R_{q}^{n\times m}$, $s_{1},s_{2}\leftarrow \chi \left(S_{\eta }^{m}\times S_{\eta }^{n}\right)$, $y_{1},y_{2}\leftarrow \chi \left(S_{\gamma -1}^{m}\times S_{\gamma -1}^{n}\right)$, and c←C. Assume that $y_{1}+cs_{1}\geq \gamma -\beta$ or $y_{2}+cs_{2}\geq \gamma -\beta$ hold. Given (A,w,c), decide whether w was generated uniformly at random from $R_{q}^{n}$ or it was generated as $w={\mathrm{Ay}}_{1}+y_{2}$.*

The advantage of adversary A in breaking the rejected Module-LWE for the set of parameters (q,n,m,γ,χ,β) can be defined as follows:

$${\mathrm{Adv}}_{\left(q,n,m,\gamma ,\chi ,\beta \right)}^{R-\mathrm{MLWE}}\left(A\right):=|{\mathrm{Game}}_{0}^{R-\mathrm{MLWE}}-{\mathrm{Game}}_{1}^{R-\mathrm{MLWE}}|.$$

${\mathrm{Game}}_{0}^{R-\mathrm{MLWE}}:=\mathrm{Pr}\left[b=1:A\leftarrow R_{q}^{n\times m},\left(s_{1},s_{2}\right)\leftarrow \chi \left(S_{\eta }^{m}\times S_{\eta }^{n}\right),\left(y_{1},y_{2}\right)\leftarrow \chi \left(S_{\gamma -1}^{m}\times S_{\gamma -1}^{n}\right),c\leftarrow C,w:={\mathrm{Ay}}_{1}+y_{2},b\leftarrow A\left(A,w,c\right)|y_{1}+cs_{1}\geq \gamma -\beta \;\mathrm{or}\;y_{2}+cs_{2}\geq \gamma -\beta \right]$

${\mathrm{Game}}_{1}^{R-\mathrm{MLWE}}:=\mathrm{Pr}\left[b=1:A\leftarrow R_{q}^{n\times m},\left(s_{1},s_{2}\right)\leftarrow \chi \left(S_{\eta }^{m}\times S_{\eta }^{n}\right),\left(y_{1},y_{2}\right)\leftarrow \chi \left(S_{\gamma -1}^{m}\times S_{\gamma -1}^{n}\right),c\leftarrow C,w\leftarrow R_{q}^{n},b\leftarrow A\left(A,w,c\right)|y_{1}+cs_{1}\geq \gamma -\beta \;\mathrm{or}\;y_{2}+cs_{2}\geq \gamma -\beta \right]$

### 3.3. Forking Lemma

The following forking lemma is adapted from [31]. *x* can be viewed as a public key of the signature scheme, and $h_{1},\ldots ,h_{q}$ can be viewed as replies to the random oracle queries.

**Lemma** **1** **(General forking lemma).** *Fix an integer q≥1 to be the number of queries. Fix set C of size |C|≥2. Let B be a randomised algorithm that takes as input $x,h_{1},\ldots ,h_{q}$, where $(h_{1},\ldots ,h_{q})\in C$, and returns a pair with the first element being index i (integer in the range {0,…,q}) and the second element being side output out. Let IG be a randomised input generation algorithm. Let the accepted probability of B be denoted as acc. This is the probability that i≠0 in the following experiment:*

- x←IG
- $h_{1},\ldots ,h_{q}\leftarrow C$
- $\left(i,out\right)\leftarrow B\left(x,h_{1},\ldots ,h_{q}\right)$

*The forking algorithm $F_{B}$ connected with B is defined in Algorithm 1.*

**Algorithm 1**
$F_{B}\left(x\right)$
1: Pick random coins ρ for B2: $h_{1},\ldots ,h_{q}\leftarrow C$3: $\left(i,out\right)\leftarrow B\left(x,h_{1},\ldots ,h_{q};\rho \right)$4: If i=0, then return (0,⊥,⊥)5: Regenerate $h_{i}^{\prime },\ldots ,h_{q}^{\prime }\leftarrow C$6: $\left(i^{\prime },out^{\prime }\right)\leftarrow B\left(x,h_{1},\ldots ,h_{i-1},h_{i}^{\prime },\ldots ,h_{q}^{\prime };\rho \right)$7: If $i=i^{\prime }$ and $h_{i}\neq h_{i}^{\prime }$, then return $\left(1,out,out^{\prime }\right)$8: Otherwise, return (0,⊥,⊥)



*Let us define the frk probability as*

$$frk=\;\mathrm{Pr}[b=1:x\leftarrow \mathrm{IG};\left(b,out,out^{\prime }\right)\leftarrow F_{B}\left(x\right)].$$

*Then,*

$$frk\geq acc\cdot \left(\frac{acc}{q}-\frac{1}{\left|C\right|}\right).$$

*Alternatively,*

$$acc\leq \frac{q}{\left|C\right|}+\sqrt{q\cdot frk}.$$

### 3.4. Lattice-Based Signature Scheme

Lattice-based cryptography is a promising candidate for the post-quantum public key cryptography standards. Among all of the submissions to the NIST PQC competition, the majority of schemes belong to the lattice-based family [17]. Many lattice-based signatures are constructed from the identification schemes using the Fiat–Shamir (FS) transform. The FS transform technique introduced in [32] allows for creating a digital signature scheme by combining an identification scheme with a hash function.

The following definition is adapted from [13].

**Definition** **5(Identification scheme)**. *An identification scheme ID is defined as a tuple of algorithms ID:=(IGen,P,C,V).*

- *The key generation algorithm IGen takes as input system parameters par and returns the public key and secret key as output (pk,sk). Public key pk defines the set of challenges C, the set of commitments W, and the set of responses Z.*
- *The prover algorithm $P=(P_{1},P_{2})$ consists of two sub-algorithms. $P_{1}$ takes as input the secret key and returns a commitment w∈W and a state st. $P_{2}$ takes as input the secret key, a commitment, a challenge, and a state and returns a response z∈Z∪{⊥}.*
- *The verifier algorithm V takes as input the public key and the conversation transcript and outputs a decision bit b=1 (accepted) or b=0 (rejected).*

In the signature scheme that uses FS transform, the signing algorithm generates a transcript (w,c,z), where a challenge *c* is derived from a commitment *w* and the message to be signed *m* as follows c:=H(w||m). The signature σ=(w,z) is valid if the transcript (w,c,z) passes the verification algorithm with b=1. The publication [33] introduced a generalisation to this technique called Fiat–Shamir, with aborts transformation that takes into consideration aborting provers.

The following signature scheme (further referred to as the basic scheme) is a slightly modified version of the scheme [34]; the description below is based on a version described in [13] (Appendix B). The signature scheme makes use of a hash function, which produces a vector of size *n* with elements in {−1,0,1} [18]. The hashing algorithm starts with applying a collision-resistant hash function (e.g., SHAKE-256) to the input to obtain a vector $s\in {\left\{0,1\right\}}^{\tau }$ from the first τ bits of the hash function’s output. Then, SampleInBall algorithm (Algorithm 1) is invoked to create a vector c in ${\left\{-1,0,1\right\}}^{n}$ with exactly τ nonzero elements. In each iteration of the for loop, the SampleInBall algorithm generates an element j∈{0,…,i} using the output of a collision-resistant hash function. Then, the algorithm performs shuffling of the elements in the vector c and takes an element from the vector s to generate −1 or 1. For an in-depth overview of the algorithm, refer to the original paper [18].


**Algorithm 2** SampleInBall.1: Initialise c as zero vector of length *n*2: for i:=n−τton−1  1: j←{0,1,…,i}  2. s←{0,1}  3. $c_{i}:=c_{j}$  4. $c_{j}:={\left(-1\right)}^{s}$
3: return c


All of the algebraic operations in the signature scheme are performed over the ring $R_{q}$. A formal definition of the key generation, signing, and verification is presented in Algorithms 3–5.


**Algorithm 3** KeyGen(par).1: $A\leftarrow R_{q}^{k\times k}$2: $s_{1},s_{2}\leftarrow S_{\eta }^{k}$3: $t:={\mathrm{As}}_{1}+s_{2}$4: **return**
$pk=\left(A,t\right),sk=\left(A,t,s_{1},s_{2}\right)$



**Algorithm 4** Sign(sk,m).1: $\left(z_{1},z_{2}\right)=\left(⊥,⊥\right)$2: **while**
$\left(z_{1},z_{2}\right)=\left(⊥,⊥\right)$ do:  1: $y_{1},y_{2}\leftarrow S_{\gamma_{1}-1}^{k}$  2. $w:={\mathrm{Ay}}_{1}+y_{2}$  3. $c:=H_{0}\left(m||w\right)\in B_{\tau }$  4. $z_{1}:=y_{1}+cs_{1}$ and $z_{2}:=y_{2}+cs_{2}$
  5. **if**
$\left|\right|z_{1}{\left|\right|}_{\infty }\geq \gamma_{1}-\beta$ or $\left|\right|z_{2}{\left|\right|}_{\infty }\geq \gamma_{1}-\beta$, then $\left(z_{1},z_{2}\right):=\left(⊥,⊥\right)$
3: **return**
$\sigma =(z_{1},z_{2},c)$



**Algorithm 5** Verify(pk,m,σ).
1:
$w^{\prime }:={\mathrm{Az}}_{1}+z_{2}-ct$
2:**if**$c=H_{0}\left(m|\right|w^{\prime }$) and $\left|\right|z_{1}{\left|\right|}_{\infty }<\gamma_{1}-\beta$ and $\left|\right|z_{2}{\left|\right|}_{\infty }<\gamma_{1}-\beta$, **return** 1 (success).3:**else**: **return** 0.



#### Correctness

Since $w={\mathrm{Ay}}_{1}+y_{2}$, $t={\mathrm{As}}_{1}+s_{2}$, $z_{1}=y_{1}+cs_{1}$. and $z_{2}=y_{2}+cs_{2}$, it holds that

$$\begin{array}{c} {\mathrm{Az}}_{1}+z_{2}-ct=A\left(y_{1}+cs_{1}\right)+\left(y_{2}+cs_{2}\right)-c\left({\mathrm{As}}_{1}+s_{2}\right)=\\ {\mathrm{Ay}}_{1}+Acs_{1}+y_{2}+cs_{2}-c{\mathrm{As}}_{1}-cs_{2}={\mathrm{Ay}}_{1}+y_{2}. \end{array}$$

Therefore, if a signature was generated correctly, it will successfully pass the verification.

### 3.5. Homomorphic Hash Function

We decided to use a homomorphic hash function instead of a homomorphic commitment scheme as in [16].

**Definition** **6(Homomorphic hash function)**. *Let + be an operation defined over X, and let ⊕ be an operation defined over R. Let $x_{1},x_{2}\in X$ be any two inputs to the hash function. A hash function f:X→R is homomorphic if it holds that*

$$f\left(x_{1}+x_{2}\right)=f\left(x_{1}\right)⊕f\left(x_{2}\right).$$

**Definition** **7(Regular hash function)**. *Let $F={\left\{f_{a}\right\}}_{a\in A}$, where $f_{a}:X\to R$ be a collection of functions indexed by a set A. A family of hash functions F is called ϵ-regular if the statistical distance between its output distribution $\left\{\left(a,f_{a}\left(x\right)\right):a\leftarrow A,x\leftarrow X\right\}$ and the uniform distribution {(a,r):a←A,r←R} is at most ϵ.*

One of the homomorphic hash functions available is called SWIFFT; it is a special case of the function proposed in [35,36,37]. SWIFFT is a collection of compression functions that are provably one-way and collision-resistant [38]. Additionally, SWIFFT has several statistical properties that can be proven unconditionally: universal hashing, regularity, and randomness extraction. However, due to the linearity, SWIFFT functions are not pseudorandom. It follows that the function is not a suitable instantiation of a random oracle [38]. Therefore, in the security proofs of the two-party signature scheme, SWIFFT is not used as a random oracle. Security proof makes use of such provable properties as regularity and collision resistance.

## 4. Proposed Two-Party Signature Scheme (DiLizium)

In the following section, we define and give detailed description of our two-party signature scheme: DiLizium. We start by defining the distributed signature scheme; the following definition is adapted from [16].

**Definition** **8(Distributed signature protocol)**. *Distributed signature protocol is a protocol between $P_{1},\ldots ,P_{n}$ parties that consists of the following algorithms:*

- *Generate public parameters par using security parameter λ as input: par← Setup($1^{\lambda }$).*
- *Each party $P_{j}$ generates a key pair consisting of secret key share and a public key using interactive algorithm and public parameters as input: $(sk_{j},pk)\leftarrow$ KeyGen ${}_{j}\left(par\right)$ for each j∈{1,…,n}.*
- *To sign a message m, each party $P_{j}$ runs an interactive signing algorithm using secret key share: (σ)← Sign ${}_{j}\left(sk_{j},m\right)$ for each j∈{1,…,n}.*
- *To verify a signature, the verifier needs to check if Verify(pk,m,σ) = 1. If the signature was generated correctly, verification should always succeed.*

### 4.1. Specification and Overview of DiLizium Signature Scheme

Table 1 describes the parameters used in the two-party signature scheme.

#### 4.1.1. Parameter Setup

Let us assume that, before starting the key generation and signing protocols, the parties invoke a Setup($1^{\lambda }$) function that, based on the security parameter λ, outputs a set of public parameters par that are described in Table 1.

#### 4.1.2. Key Generation

$H_{1}$ and $H_{2}$ are some collision-resistant hash functions. The key generation protocol is parametrised by the set of public parameters par. The client begins the key generation process by sampling a share of matrix $A_{c}$ and by sending out the commitment to this share $hk_{c}=H_{1}\left(A_{c}\right)$. The server generates its matrix share $A_{s}$ and sends commitment $hk_{s}$ to the client. Upon receiving commitments, the client and server exchange matrix shares and check if the openings for the commitments were correct. If openings are successfully verified, the client and server locally compute composed matrix $A=A_{c}+A_{s}$.

The client proceeds by generating two secret vectors ($s_{1}^{c}$, $s_{2}^{c}$) and by computing its share of the public key $t_{c}={\mathrm{As}}_{1}^{c}+s_{2}^{c}$. The client sends out a commitment to the public key share $comk_{c}=H_{2}\left(t_{c}\right)$. The server samples its secret vectors ($s_{1}^{s}$, $s_{2}^{s}$) and uses them to compute its public key share $t_{s}$. Next, the server sends commitment to the public key share $comk_{s}$ to the client.

Once the client and server have received commitments from each other, the client and server exchange public key shares. Next, the client and server both locally check if the commitments were opened correctly. If these checks succeed, the client and server locally compute the composed public key $t=t_{c}+t_{s}$. The final public key consists of composed matrix A and vector t.

It is necessary to include the server’s public key share $t_{s}$ to the client’s secret key $sk_{c}$ and vice versa. During the signing process, the client needs to use the server’s public key share to verify the correctness of a commitment.

Protocol 1 describes two-party key generation between the parties in a more formal way. Instructions of the protocol are the same for the client and server. Therefore, Protocol 1 presents the behavior of the *n*th party, n∈{c,s}.

#### 4.1.3. Signing

HomH is a homomorphic hash function from the SWIFFT family. $H_{0}$ is a hash function that outputs a vector of length *n* with exactly τ coefficients being either −1 or 1 and the rest being 0 as described in Algorithm 2. $H_{3}$ is a collision-resistant hash function.

The client starts the signing process by generating its shares of masking vectors ($y_{1}^{c},y_{2}^{c}$) and by computing a share of w. Next, the client uses a homomorphic hash function to compute $com_{c}=HomH\left(w_{c}\right)$ and hashes it using some collision-resistant hash function $h_{c}=H_{3}\left(com_{c}\right)$. The composed output of the homomorphic hash function $com=com_{c}+com_{s}$ is later used to derive a challenge. Therefore, it is crucial to ensure that $com_{c},com_{s}$ have not been chosen maliciously. Thus, before publishing these shares, the client and server should exchange commitments to the shares $h_{c},h_{s}$.

The server, in turn, generates its shares of masking vectors ($y_{1}^{s},y_{2}^{s}$), computes its share of w, and sends commitment to $com_{s}=HomH\left(w_{s}\right)$. After receiving commitments $h_{c},h_{s}$ from each other, the client and server open the commitments by sending out shares $com_{s},com_{c}$.

The client proceeds by checking if the server opened its commitment correctly. If the check succeeds, the client computes $com=com_{c}+com_{s}$ and derives challenge $c=H_{0}\left(m||com\right)$. Next, the client computes potential signature shares $\left(z_{1}^{c},z_{2}^{c}\right)$ and performs rejection sampling. If all of the conditions in rejection sampling are satisfied, the client sends its signature share to the server.

The server checks if the client opened its commitment correctly. If the check succeeds, the server computes composed com and derives challenge *c*. Next, the server computes its potential signature shares $\left(z_{1}^{s},z_{2}^{s}\right)$ and performs rejection sampling. If all of the conditions in rejection sampling are satisfied, the server sends its signature share to the client.

Finally, the client performs verification if $com_{s}$ indeed contains $w_{s}$. The client reconstructs $w_{s}$ as ${\mathrm{Az}}_{1}^{s}+z_{2}^{s}-ct_{s}$ and checks if it is a valid opening for $com_{s}$. If the check succeeds, the client computes the final signature $\left(z_{1},z_{2}\right)$. The server performs the same verification that $com_{c}$ indeed contains $w_{c}$ using $\left(z_{1}^{c},z_{2}^{c}\right)$ and $t_{c}$. If the check succeeds, the server computes and outputs the final signature.

Protocol 2 describes the two-party signing process in the more formal way.

#### 4.1.4. Verification

Verification is almost the same as in the original scheme except the verifier needs to apply homomorphic hash function on the reconstructed $w^{\prime }$ in order to check the correctness of challenge. Algorithm 6 describes verification in the more formal way.

#### 4.1.5. Correctness

Since $w={\mathrm{Ay}}_{1}+y_{2}$, $t={\mathrm{As}}_{1}+s_{2}$, $z_{1}=y_{1}+cs_{1}$ and $z_{2}=y_{2}+cs_{2}$ it holds that:

${\mathrm{Az}}_{1}+z_{2}-ct=A\left(y_{1}+cs_{1}\right)+\left(y_{2}+cs_{2}\right)-c\left({\mathrm{As}}_{1}+s_{2}\right)={\mathrm{Ay}}_{1}+Acs_{1}+y_{2}+cs_{2}-c{\mathrm{As}}_{1}-cs_{2}={\mathrm{Ay}}_{1}+y_{2}$.

Furthermore, by triangle inequality, it holds that if $\left|\right|z_{1}^{s}{\left|\right|}_{\infty }<\gamma -\beta$ and $\left|\right|z_{1}^{c}{\left|\right|}_{\infty }<\gamma -\beta$, then $\left|\right|z_{1}{\left|\right|}_{\infty }=\left|\right|z_{1}^{s}+z_{1}^{c}{\left|\right|}_{\infty }<||z_{1}^{s}{\left|\right|}_{\infty }+\left|\right|z_{1}^{c}{\left|\right|}_{\infty }=2\gamma -2\beta$. The same holds for the second signature component $z_{2}$. This means that $\gamma_{2}$ can be defined as $\gamma_{2}=2\gamma$ and $\beta_{2}=2\beta$. Therefore, if a signature was generated correctly, verification always succeed.


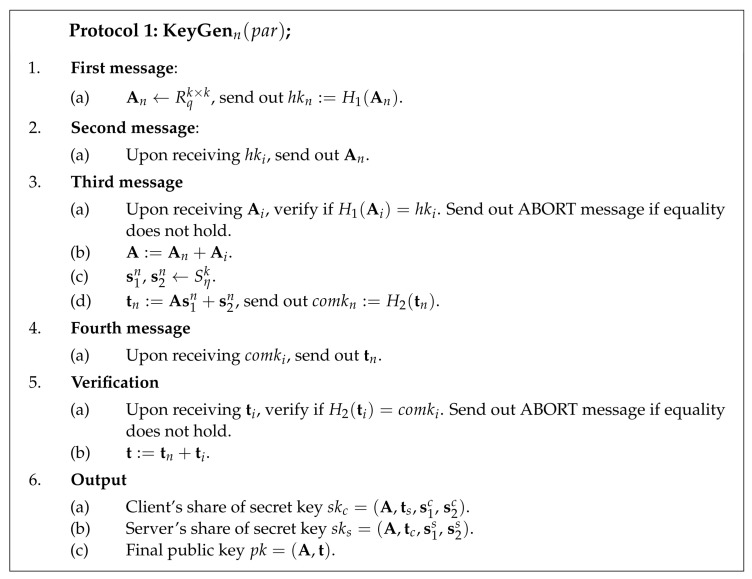



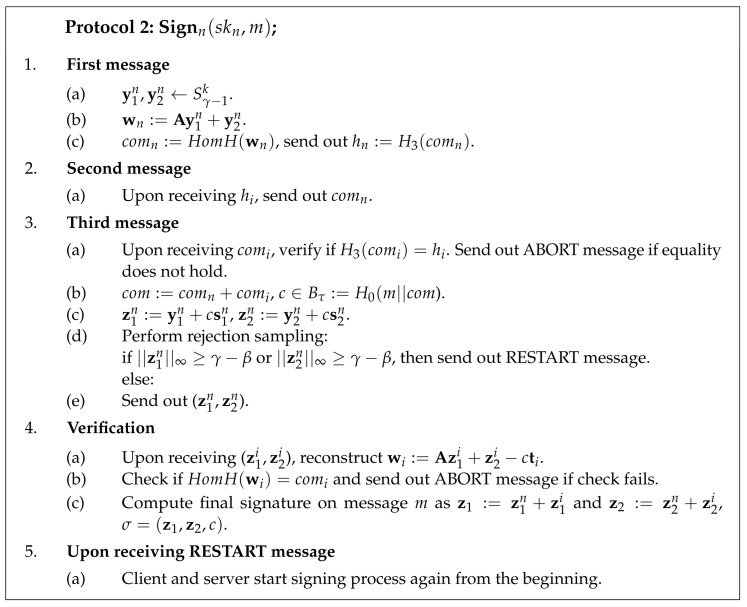



**Algorithm 6** Verify(pk,σ,m)1: Compute $w^{\prime }:={\mathrm{Az}}_{1}+z_{2}-ct$.2: **if**
c= H($m||HomH(w^{\prime })$) and $\left|\right|z_{1}{\left|\right|}_{\infty }<\gamma_{2}-\beta_{2}$ and $\left|\right|z_{2}{\left|\right|}_{\infty }<\gamma_{2}-\beta_{2}$: **return** 1 (success).3: **else**: **return** 0.


*Security*

**Definition** **9 (Existential Unforgeability under Chosen Message Attack).** *The distributed signature protocol is Existentially Unforgeable under Chosen Message Attack (DS-UF-CMA) if, for any probabilistic polynomial time adversary A, its advantage of creating successful signature forgery is negligible. The advantage of adversary is defined as the probability of winning in the experiment ${\mathrm{Exp}}^{\mathrm{DS}-\mathrm{UF}-\mathrm{CMA}}$:*

$${\mathrm{Adv}}^{\mathrm{DS}-\mathrm{UF}-\mathrm{CMA}}\left(A\right):=\mathrm{Pr}\left[{\mathrm{Exp}}^{\mathrm{DS}-\mathrm{UF}-\mathrm{CMA}}\left(A\right)\to 1\right].$$

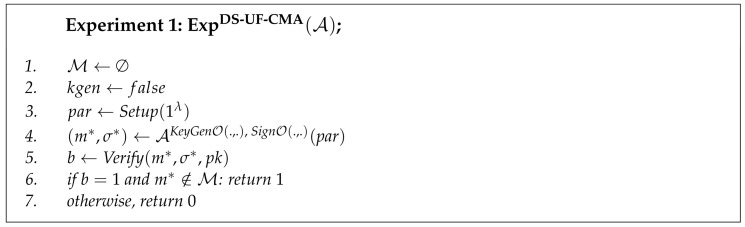



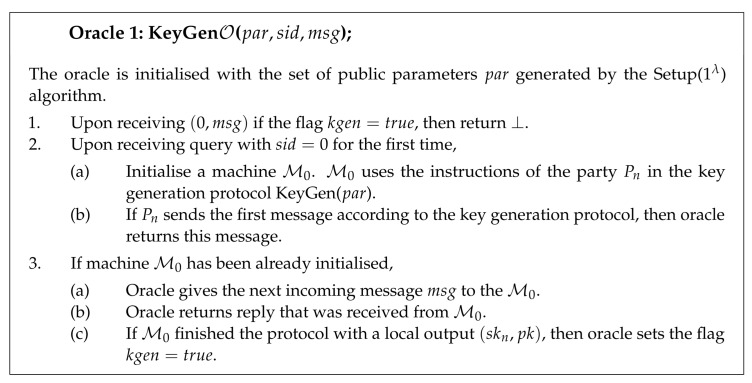



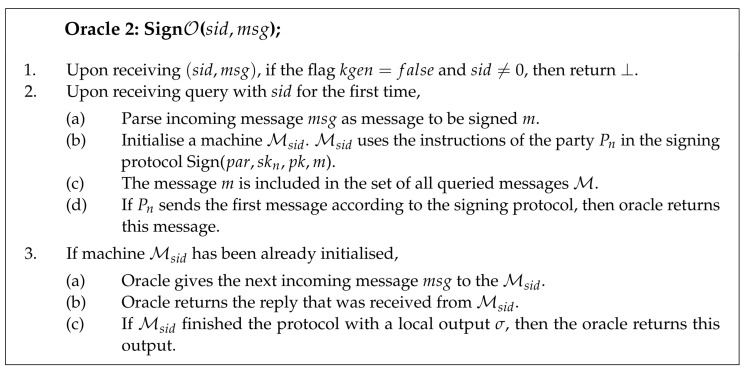

**Theorem** **1.** *Assume a homomorphic hash function $HomH:{\left\{0,1\right\}}^{a\cdot b}\to Z_{p}^{b}$ is provably collision-resistant and ϵ-regular then for any probabilistic polynomial time adversary A that makes a single query to the key generation oracle; $q_{s}$ queries to the signing oracle; and $q_{h}$ queries to the random oracles $H_{0},H_{1},H_{2},$ and $H_{3}$, the distributed signature protocol is DS-UF-CMA secure in the random oracle model under Module-LWE, rejected Module-LWE, and Module-SIS assumptions.*

This section presents the main idea for the security proof of the proposed scheme; the full proof is given in Appendix A. The proof considers only the classical adversary and relies on the forking lemma. The idea of the proof is, given an adversary A that succeeds in creating forgeries for the distributed signature protocol, to construct an algorithm around it that can be used to solve Module-SIS problem or to break the collision resistance of the homomorphic hash function. The idea of our proof relies on the proofs from [7,16,31,39].

The proof consists of two major steps. In the first step, we construct a simulator B. Algorithm B is constructed such that it fits all of the assumptions of the forking lemma. B simulates the behavior of a single honest party $P_{n}$ without using its actual secret key share. In the second step, the forking algorithm is invoked to obtain two forgeries with distinct challenges and the same commitments.

##### 4.1.6. Simulation

In the key-generation process, we need to simulate the way the matrix share $A_{n}$ and the public vector share $t_{n}$ are constructed. Due to the use of random oracle commitments, once the simulator obtains the adversary’s commitment $hk_{i}$, it can extract the matrix share $A_{i}$. Next, the simulator computes its matrix share $A_{n}:=A-A_{i}$ using a resulting random matrix $A\in R_{q}^{k\times k}$ and programs random oracle $H_{1}\left(A_{n}\right):=hk_{n}$.

Due to the Module-LWE assumption, the public vector share of the honest party $t_{n}$ is indistinguishable from the uniformly random vector sampled from the ring $R_{q}^{k}$. Using the same strategy as that for the matrix share, the simulator sets it public vector share $t_{n}:=t-t_{i}$ after seeing the adversary’s commitment and programs the random oracle $H_{2}\left(t_{n}\right):=comk_{n}$.

The signature share generation starts with choosing a random challenge c∈C from the set of all possible challenges. Then, the simulator proceeds with randomly sampling two signature shares from the set of all possible signature shares $z_{1}^{n},z_{2}^{n}\leftarrow S_{\gamma -\beta -1}^{k}$. The share of vector w is computed from the signature shares, public vector share, and challenge as $w_{n}={\mathrm{Az}}_{1}^{n}+z_{2}^{n}-ct_{n}$. Then, the simulator extracts value $com_{i}$ from the adversary’s commitment $h_{i}$, computes the composed value com, and programs random oracle $H_{0}\left(com||m\right):=c$.

##### 4.1.7. Forking Lemma

The combined public key consists of matrix A uniformly distributed in $R_{q}^{k\times k}$ and vector t uniformly distributed in $R_{q}^{k}$. We want to replace it with the Module-SIS instance $[\begin{array}{c} A^{\prime }|I \end{array}]$, where $A^{\prime }\in R_{q}^{k\times (k+1)}$. The view of adversary does not change if we set $A^{\prime }= [\begin{array}{c} A|t \end{array}]$.

In order to conclude the proof, we need to invoke the forking lemma to receive two valid forgeries from the adversary that are constructed using the same commitment $com=com^{\prime }$ but different challenges $c\neq c^{\prime }$. Using these forgeries, it is possible to find a solution to the Module-SIS problem on input $A^{\prime }= [\begin{array}{c} A|t \end{array}]$ or to break the collision resistance of the homomorphic hash function.

As both forgeries $out=(com,c,z_{1},z_{2},m)$ and $out^{\prime }=\left(com^{\prime },c^{\prime },z_{1}^{\prime },z_{2}^{\prime },m^{\prime }\right)$ are valid, it holds that

$$HomH\left({\mathrm{Az}}_{1}+z_{2}-ct\right)=com=com^{\prime }=HomH\left({\mathrm{Az}}_{1}^{\prime }+z_{2}^{\prime }-c^{\prime }t\right)$$

If ${\mathrm{Az}}_{1}+z_{2}-ct\neq {\mathrm{Az}}_{1}^{\prime }+z_{2}^{\prime }-c^{\prime }t$, then we found a collision for the homomorphic hash function. If ${\mathrm{Az}}_{1}+z_{2}-ct={\mathrm{Az}}_{1}^{\prime }+z_{2}^{\prime }-c^{\prime }t$, then it can be rearranged as ${\mathrm{Az}}_{1}-{\mathrm{Az}}_{1}^{\prime }+z_{2}-z_{2}-ct+c^{\prime }t=0$ and this in turn leads to

$$[\begin{array}{c} A|I|t \end{array}] [\begin{array}{c} z_{1}-z_{1}^{\prime }\\ z_{2}-z_{2}^{\prime }\\ c^{\prime }-c \end{array}]=0$$

Considering that $[\begin{array}{c} A|I|t \end{array}]$ is an instance of Module-SIS problem, we found a solution for Module-SIS with parameters (q,k,k+1,ξ), where $\xi \leq 2(\gamma_{2}-\beta_{2})$.

## 5. Performance

In this section, we analyse the performance of our scheme according to the following metrics:

- Number of communication rounds in key generation and signing protocols,
- Keys and signature sizes, and
- Number of rejection sampling rounds.

It should be noted that this section does not present the exact parameter choice for the scheme and does not argue the bit security of the scheme for these parameters. The parameter choice presented in this section is illustrative and is given to provide performance estimations of the proposed scheme. Choosing correct parameters for post-quantum schemes is a nontrivial multidimensional optimisation task as parameters should be chosen such that the scheme has the small signature and key sizes while having enough bits of security and an optimal number of communication rounds. Additionally, the security of the proposed scheme relies on rejected Module-LWE, which is not a well-studied assumption, and therefore, it is difficult to estimate the bit security of the proposed scheme. The parameters presented in Table 2 are chosen based on parameters proposed in Crystals-Dilithium [18] so that the expected number of repetitions of the signing process is practical.

### 5.1. Number of Rejection Sampling Rounds

To estimate the number of rejection sampling rounds in the signing process, it is necessary to compute the probability that the following holds for both parties: $\left|\right|z_{1}^{n}{\left|\right|}_{\infty }<\gamma -\beta$ and $\left|\right|z_{2}^{n}{\left|\right|}_{\infty }<\gamma -\beta$. Let σ be a coefficient of $cs_{i}^{n}$. If coefficients of $y_{i}^{n}$ are in the range {−γ+β+1−σ,…,γ−β−1−σ}, then the corresponding coefficients of $z_{i}^{n}$ are in the range {−γ+β+1,…,γ−β−1}. Therefore, the size of the correct coefficient range for $y_{i}^{n}$ is 2(γ−β)−1 and the coefficients of $y_{i}^{n}$ have 2γ−1 possibilities. Then, the probability that every coefficient of $y_{i}^{n}$ in the correct range is as follows:

$${\left(\frac{2(\gamma -\beta )-1}{2\gamma -1}\right)}^{n\cdot k}$$

As the client and server sample vectors $y_{i}^{n}$ independently in the beginning of the signing protocol, the probability that the check succeeds for both signature components on the client and server side is the following:

$$\mathrm{Pr}[\mathrm{success}]={\left(\frac{2(\gamma -\beta )-1}{2\gamma -1}\right)}^{n\cdot k\cdot 4}$$

The expected number of repetitions can be estimated as E=1/Pr[success].

### 5.2. Signature and Key Sizes

The **public key** consists of two components: matrix $A\in R_{q}^{k\times k}$ and vector $t\in R_{q}^{k}$. The matrix A can be generated out of 256-bit seed using an extendable output function, as proposed in the Crystals-Dilithium signature scheme [18]. While using this approach, only the seed used to generate the matrix needs to be stored. As both parties need to generate their matrix share, two seeds should be stored to represent matrix A. Each seed is converted to the matrix form using an extendable output function; after that, two matrix shares can be added together. The size of the public key in bytes is as follows:

$$\frac{2\cdot 256+n\cdot k\cdot \lceil \log(q)\rceil }{8}.$$

The **secret key** of the party $P_{n}$ consists of two vectors $s_{1}^{n}$, $s_{2}^{n}\in S_{\eta }^{k}$, matrix A, and vector $t_{i}\in R_{q}^{k}$. It should be noted that vectors $s_{1}^{n}$, $s_{2}^{n}$ may contain negative values as well, so one bit should be reserved for each coefficient to indicate the sign. Therefore, the size of the secret key in bytes can be computed as follows:

$$\frac{2\cdot n\cdot k\cdot (\lceil \log(\eta )\rceil +1)+2\cdot 256+n\cdot k\cdot \lceil \log(q)\rceil }{8}=\frac{n\cdot k\cdot (2\cdot (\lceil \log(\eta )\rceil +1)+\lceil \log(q)\rceil )+2\cdot 256}{8}.$$

Finally, a **signature** consists of three components: $z_{1},z_{2}\in S_{\gamma_{2}-\beta_{2}-1}^{k}$, and $c\in {\left\{0,1\right\}}^{n}$ with exactly τ coefficients being either −1 or 1 and the rest being 0. All of the components may contain negative values, so for each coefficient of $z_{1},z_{2},$*c* one bit should be reserved to indicate the sign. With regard to storing *c*, it is possible to store only the positions of −1 and 1 in *c*. Therefore, the size of the signature in bytes can be computed as

$$\frac{2\cdot n\cdot k\cdot \left(\left\lceil \log\left(\gamma_{2}-\beta_{2}-1\right)\right\rceil +1\right)+\tau \cdot \left(\left\lceil \log\left(n\right)\right\rceil +1\right)}{8}.$$

In order to better understand key and signature sizes, let us assume the choice of parameters defined in Table 2. The key and signature sizes corresponding to this choice of parameters are listed in Table 3.

### 5.3. Communication between Client and Server

In order to generate a key pair, four rounds of communication between the client and server are needed. Table 4 shows the sizes of messages that are exchanged between the client and server during the key generation process using illustrative parameters from Table 2. The first message is output of a hash function (commitment to matrix $A_{i}$), which consists of 256 bits. The second message contains not the matrix share itself but the seed of 256 bits that was use to generate it. The third message is output of a hash function (commitment to vector $t_{i}$), which consists of 256 bits. The fourth message is the share of public key $t_{i}$, the size of which is $\frac{n\cdot k\cdot \lceil \log(q)\rceil }{8}$ bytes.

The number of communication rounds during the signing process depends on the number of rejections *E*. If there are no rejections, the signature generation process requires three rounds of communication between the client and server. For *E* rejections, the number of communication rounds equals to 2E+1. Table 5 shows the sizes of messages exchanged between the client and server during the signing process using illustrative parameters from Table 2. The first message is output of a hash function (commitment to $com_{i}$), which consists of 256 bits. The size of the second message in the signing process $com_{i}$ is caused by the structure of SWIFFT hash function. To calculate $HomH(w_{i})$, the vector $w_{i}$ that consists of nk elements is divided into 15 input blocks of 256 bytes each. The output produced by the homomorphic hash function consists of 15 blocks of 128 bytes each. The third message consists of the signature shares $\left(z_{1}^{i},z_{2}^{i}\right)$, the size of which is $\frac{2\cdot n\cdot k\cdot (\left\lceil \log\left(\gamma_{2}-\beta_{2}-1\right)\right\rceil +1)}{8}$ bytes.

## 6. Comparison to Prior Work

Table 6 presents the comparison of our scheme DiLizium with other lattice-based threshold signature schemes [16,27,40]. Column “Rounds” shows the number of communication rounds in signing protocol; for the schemes with rejection sampling, it is assumed that the rejection sample passes from the first attempt. We also provide a more detailed comparison with [16], due to the fact both works are based on variants of Crystals-Dilithium and have a similar structure. We leave out the comparison with publications [21,22,23,24,25,26] as these discuss multisignatures instead of threshold.

The threshold signature schemes from [16] are based on Dilithium-G, which is a version of Crystals-Dilithium that uses sampling from a discrete Gaussian distribution for the generation of secret vectors. The usage of Gaussian distribution helps to decrease the number of rejections in signature schemes that follow the FSwA paradigm [16]. However, the implementation of sampling from a discrete Gaussian distribution in a manner secure against side-channel attacks is considered difficult. Therefore, in our scheme, we decided to use sampling from a uniform distribution.

Due to the structure of our scheme, we use a non-standard security assumption that was introduced in [26]. In future work, we aim to modify security proof such that it will no longer be needed to rely on rejected Module-LWE. The security of threshold signature scheme from [16] relies only on standard problem: Module-LWE and Module-SIS.

Additionally, we compare the message sizes of Dåmgard et al. [16] $DS_{3}$ with our scheme. Since this paper [16] does not provide an instantiation of parameters, we use the recommended parameters for Dilithium-G from [29] for the signature scheme. We only changed the modulus *q*, since by Theorem 4 [16], *q* should satisfy q=5mod8. We selected parameters for the homomorphic commitment scheme based on the third parameter set from Baum et al. [42] (Table 2) such that the conditions from Lemma 5 and Lemma 7 are satisfied. Table 7 and Table 8 present parameters that are needed to compute message sizes. We only provide a comparison of messages sent during the signing process because messages exchanged during the key generation process are similar in both schemes (Table 9).

The first message is the output of a hash function (commitment to $com_{n}$), which consists of 256 bits. The second message is a commitment $com_{n}$. The homomorphic commitment scheme defined in Dåmgard et al. [16] (Figure 7) describes a commitment to a single ring element $w\in R_{q}$. In order to commit to a vector $w\in R_{q}^{k}$, it is proposed to commit to each vector element separately. Therefore, the byte size of the second message can be computed as $\frac{k_{sig}\cdot N\cdot \left(m+k_{com}\right)\cdot \left\lceil \log\left(q_{com}\right)\right\rceil }{8}$. The third message consists of a signature share $z_{n}$ and an opening for the commitment $r_{n}$. We know that, for a valid signature share, it holds that $\left|\right|z_{n}{\left|\right|}_{2}\leq B_{sig}$ and we know that ${\left||x|\right|}_{\infty }\leq {\left||x|\right|}_{2}$. The signature share may contain negative values, so for each coefficient of $z_{n}$, one bit should be reserved to indicate the sign. Therefore, the approximate byte size of the signature share is $\frac{n\cdot \left(l+k_{sig}\right)\cdot \left(\left\lceil \log\left(B_{sig}\right)\right\rceil +1\right)}{8}$. For a valid opening, it holds that $\left|\right|r_{n}{\left|\right|}_{2}\leq B_{com}$. The value $r_{n}$ also may contain negative values, so for each coefficient of $r_{n}$, one bit should be reserved to indicate the sign. The approximate byte size of the commitment opening is $\frac{k_{sig}\cdot N\cdot m^{\prime }\cdot \left(\left\lceil \log\left(B_{com}\right)\right\rceil +1\right)}{8}$.

From Table 9, we can see that the size of the second and the third messages in Dåmgard et al.’s $DS_{3}$ scheme is much larger than that in DiLizium. The reason is that scheme $DS_{3}$ Dåmgard et al. uses lattice-based homomorphic commitments. For the signature scheme to be secure, it was required to have statistical binding. Parameters that guarantee statistical binding are not very practical; however, there may exist optimal parameter choice.

Currently, it is not possible to provide a more detailed comparison of the efficiency of these schemes. The main reason is that neither of the works have a reference implementation yet. Therefore, we leave the implementation of the proposed scheme and a detailed comparison for future research.

## 7. Conclusions

Nowadays, threshold signature schemes have a variety of practical applications. There are several efficient threshold versions of the RSA and (EC)DSA signature schemes that are used in practice. However, threshold instantiations of post-quantum signature schemes are less researched. Previous researches have demonstrated that creating threshold post-quantum signatures is a highly non-trivial task. Some of the proposed schemes yield inefficient implementation, while others have incomplete security proofs.

In this work, we presented a new lattice-based two-party signature scheme: DiLizium. Our construction uses the SWIFFT homomorphic hash function to compute commitment in the signing process. We provide security proof for our scheme in the classical random oracle model under the Module-LWE, rejected Module-LWE, and Module-SIS assumptions. The proposed scheme can potentially substitute distributed RSA and ECDSA signature schemes in authentication applications such as Smart-ID [11]. This would allow for using these applications even in the quantum computing era.

Compared with the scheme proposed in [16], this work does not use sampling from the discrete Gaussian distribution and does not use a lattice-based homomorphic commitment scheme. In the key generation and signing processes, our scheme uses uniform sampling, which facilitates secure implementations in the future.

The security proof of the proposed scheme is based on non-standard security assumption: rejected Module-LWE. Removing this assumption from the security proof is an important part of future work. Furthermore, the concept of homomorphic hash functions is new and has not been properly studied yet. We aim to research the properties and usage of homomorphic hash functions more deeply in future work. The implementation of the proposed scheme and the exact choice of parameters for this implementation is left for future research, which may also involve optimisation of the size of keys and signature, and security proof against the quantum adversary.

## Figures and Tables

**Table 1:** Parameters for the two-party protocol.

Parameter	Description
*n*	degree bound of the polynomials in the ring
*q*	modulus
(k,k)	dimension of matrix and vectors used in the scheme
γ	size bound of the coefficients in the masking vector share
$\gamma_{2}$	size bound of coefficients in the composed masking vector
η	size bound of coefficients in the secret key share
τ	number on nonzero elements in the output of special hash function $H_{0}$
β	maximum possible coefficient of the client’s and server’s shares of $cs_{i}$, where i∈{1,2}
$\beta_{2}$	maximum possible coefficient of $cs_{i}$, where i∈{1,2}
(a,b,p)	parameters for the homomorphic hash function: a·b is the input length, *b* is the output length, and *p* is the modulus

**Table 2:** Illustrative parameters.

Parameters	Sizes
*n*	256
*q*	8,380,417
(k,k)	(5, 5)
γ	$2^{19}$
$\gamma_{2}$	$2^{20}$
η	2
τ	60
β	120
$\beta_{2}$	240
(a,b,p)	(64,16,257)

**Table 3:** Key and signature sizes and expected number of repetitions of the signing protocol.

Values	Sizes
Public key pk	3744 bytes
Secret key share $sk_{i}$	4384 bytes
Signature σ	6788 bytes
Expected number of repetitions	3.23

**Table 4:** Message sizes in the key generation process.

Messages	Sizes
First message $hk_{i}$	256 bits
Second message $A_{i}$ (as seed)	256 bits
Third message $comk_{i}$	256 bits
Fourth message $t_{i}$	3680 bytes

**Table 5:** Message sizes in the signing process.

Messages	Sizes
First message $\left(h_{i}\right)$	256 bits
Second message $com_{i}$	1920 bytes
Third message $\left(z_{1}^{i},z_{2}^{i}\right)$	6080 bytes

**Table 6:** Comparison with prior work.

	Functionality	Paradigm	Rounds	Security
Bendlin et al. [27]	*t*-out-of-*n*	Hash-and-Sign	1	Gentry et al. [41]
Boneh et al. [40]	*t*-out-of-*n*	Any (Universal Thresholdizer)	1	LWE
Dåmgard et al. [16] $DS_{2}$	*n*-out-of-*n*	FSwA	2	MLWE
Dåmgard et al. [16] $DS_{3}$	*n*-out-of-*n*	FSwA	3	MLWE, MSIS
Our protocol (DiLizium)	2-out-of-2	FSwA	3	MLWE, MSIS, R-MLWE

**Table 7:** Illustrative parameters for Dåmgard et al. $DS_{3}$ [16].

Parameters for Signature Scheme	Description	Sizes
*n*	The degree bound of the polynomials in the ring	256
$q_{sig}$	Modulus	8,380,781
$\left(k_{sig},l\right)$	Dimension of matrix and vectors	(4, 4)
η	Size bound of coefficients in the secret key share	5
τ	Number on non-zero elements in the output of hash function $H_{0}$	60
$\sigma_{sig}$	Standard deviation of the Gaussian distribution	≈17,900
$B_{sig}$	The maximum $l_{2}$ norm of signature share $z_{j}\in R_{q_{sig}}^{l+k_{sig}}$	≈990,000

**Table 8:** Illustrative parameters for Dåmgard et al. [16] statistically binding commitment scheme.

Parameters for Commitment Scheme	Description	Sizes
*N*	The degree bound of the polynomials in the ring	1024
$q_{com}$	Modulus	≈2${}^{55}$
$\left(m,m^{\prime },k_{com}\right)$	Dimension of matrices and vectors	(6, 9, 1)
$\sigma_{com}$	Standard deviation of the Gaussian distribution	≈46,000
$B_{com}=4\sigma_{com}\sqrt{m^{\prime }N}$	The maximum $l_{2}$ of commitment opening $r_{j}\in R_{q_{com}}^{m^{\prime }}$	≈17,664,000

**Table 9:** Illustrative comparison of DiLizium and Dåmgard et al. $DS_{3}$ message sizes.

Messages	DiLizium	Dåmgard et al. ${\mathrm{DS}}_{3}$
First message	256 bits	256 bits
Second message	1920 bytes	197,120 bytes
Third message	6080 bytes	125,184 bytes

## Abbreviations

The following abbreviations are used in this manuscript:

RSA	Rivest–Shamir–Adleman cryptosystem
DSA	Digital Signature Algorithm
ECDSA	Elliptic Curve Digital Signature Algorithm
NIST	National Institute of Standards and Technology
PQC	Post-Quantum Cryptography
QSCD	Qualified Electronic Signature Creation Device
LWE	Learning with Errors
SIS	Short Integer Solution
FSwA	Fiat–Shamir with Aborts
MPC	Multiparty Computation
GPV	Gentry–Peikert–Vaikuntanathan
FS	Fiat-Shamir
SHAKE	Secure Hash Algorithm and KECCAK
DS-UF-CMA	Distributed Signature Unforgeability Against Chosen Message Attacks
naHVZK	no-abort Honest-Verifier Zero-Knowledge

## Appendix A. Full Security Proof

This section presents detailed security proof for the two-party signature scheme.

**Definition** **A1****(no-abort Honest-Verifier Zero-Knowledge).**
*An identification scheme is said to be $\epsilon_{ZK}$-naHVZK if there exists a probabilistic expected polynomial-time algorithm Sim that is given only the public key pk and that outputs (w,c,z) such that the following holds:*

- *The distribution of the simulated transcript produced by Sim ((w,c,z)←Sim(pk)) has a statistical distance at most $\epsilon_{ZK}$ from the real transcript produced by the transcript algorithm $\left(w^{\prime },c^{\prime },z^{\prime }\right)\leftarrow Trans\left(sk\right)$.*
- *The distribution of c from the output (w,c,z)←Sim(pk) conditioned on c≠⊥ is uniformly random over the set C.*

**Theorem** **A1.**
*Assume a homomorphic hash function $HomH:{\left\{0,1\right\}}^{a\cdot b}\to Z_{p}^{b}$ is provably collision-resistant and ϵ-regular; then for any probabilistic polynomial time adversary A that makes a single query to the key generation oracle, $q_{s}$ queries to the signing oracle, and $q_{h}$ queries to the random oracles $H_{0},H_{1},H_{2},H_{3}$, the distributed signature protocol is DS-UF-CMA secure in the random oracle model under Module-LWE, rejected Module-LWE, and Module-SIS assumptions.*

**Proof.** Given an adversary A that succeeds in breaking the distributed signature protocol with advantage ${\mathrm{Adv}}^{\mathrm{DS}-\mathrm{UF}-\mathrm{CMA}}\left(A\right)$, a simulator B is constructed. B simulates the behaviour of the single honest party without using honestly generated secret keys for the computation. Algorithm B is constructed such that it fits all the assumptions of the forking lemma. By the definition of forking algorithm, it was required that B is given a public key and a random oracle query replies as input. B simulates the behaviour of the honest party $P_{n}$, and the party $P_{i}$ is corrupted by the adversary. The algorithm B is defined in Algorithm A1.

**Algorithm A1**B($pk,h_{1},\ldots ,h_{q_{h}+q_{s}+1}$).
1: Create empty hash tables $HT_{i}$ for i∈{0,…,3}.
2: Create a set of queried messages M=∅.
3: Simulate the honest party oracle as follows: - Upon receiving a query from A of the form (sid,msg), reply to the query as described in $SimO_{KeyGen}$ (Oracle A1) and $SimO_{Sign}$ (Oracle A2). - If one of the oracles terminates with output of the form (0,⊥), then B also terminates with the same output (0,⊥).
4: Simulate random oracles as follows: - Upon receiving a query from A to the random oracle, reply to the query as described in Algorithm A2.
5: Upon receiving a forgery $\sigma =(z_{1},z_{2},c)$ on message $m^{\prime }$ from A: - If $m^{\prime }\in M$, then B terminates with output (0,⊥). - Compute $com^{\prime }:=HomH\left({\mathrm{Az}}_{1}+z_{2}-ct\right)$. - Make query $c^{\prime }\leftarrow H_{0}(m^{\prime }||com^{\prime })$. - If $c\neq c^{\prime }$ or $\left|\right|z_{1}{\left|\right|}_{\infty }\geq \gamma_{2}-\beta_{2}$ or $\left|\right|z_{2}{\left|\right|}_{\infty }\geq \gamma_{2}-\beta_{2}$, then B terminates with output (0,⊥). - Find index $i_{f}\in \left[q_{h}+q_{s}+1\right]$ such that $c^{\prime }=h_{i_{f}}$. B terminates with the output $\left(i_{f},out=\left(com^{\prime },c^{\prime },z_{1},z_{2},m^{\prime }\right)\right)$

### Appendix A.1. Random Oracle Simulation

There are several random oracles that need to be simulated:

1. $H_{0}:{\left\{0,1\right\}}^{*}\to C$
  [*C* is a set of all vectors in ${\left\{-1,0,1\right\}}^{n}$ with exactly τ nonzero elements]
2. $H_{1}:{\left\{0,1\right\}}^{*}\to {\left\{0,1\right\}}^{l_{1}}$
3. $H_{2}:{\left\{0,1\right\}}^{*}\to {\left\{0,1\right\}}^{l_{2}}$
4. $H_{3}:{\left\{0,1\right\}}^{*}\to {\left\{0,1\right\}}^{l_{3}}$

All of the random oracles are simulated as described in Algorithm A2. Additionally, there is a searchHash(HT,h) algorithm for searching entries from the hash table defined in Algorithm A3.

**Algorithm A2**$H_{i}\left(x\right)$.
$HT_{i}$ is a hash table that is initially empty.
1: On a query *x*, return element $HT_{i}\left[x\right]$ if it was previously defined.
2: Otherwise, sample output *y* uniformly at random from the range of $H_{i}$ and return $HT_{i}\left[x\right]:=y$

**Algorithm A3** searchHash(HT,h)
1: For value *h*, find its preimage *m* in the hash table such that HT[m]=h.
2: If preimage of value *h* does not exist, set flag alert and set preimage m=⊥.
3: If for value *h* more than one preimage exists in hash table HT, set flag bad.
4: **Output:** (m,alert,bad)

Simulators for the key generation and signing processes were constructed using several intermediate games. The goal was to remove the usage of the actual secret key share of the party $P_{n}$ from both processes. Let Pr[$G_{i}$] denote the probability that B does not output (0,⊥) in the game $G_{i}$. This means that the adversary must have created a valid forgery (as defined in Algorithm A1). Then, Pr[$G_{0}$] $={\mathrm{Adv}}^{\mathrm{DS}-\mathrm{UF}-\mathrm{CMA}}\left(A\right)$. In Game 0, B simulates the honest party behaviour using the same instructions as in the original KeyGen ${}_{n}\left(par\right)$ and Sign ${}_{n}\left(sk_{n},m\right)$ protocols.

### Appendix A.2. Game 1

In Game 1, only signing process ics changed with respect to the previous game. The simulator for the signing process in Game 1 is described in Algorithm A4. Challenge *c* is now sampled uniformly at random, and the signature shares are computed without communicating with the adversary. Changes with respect to the previous game are highlighted.

**Algorithm A4**$Sim_{Sign}\left(sk_{n},pk,m\right)$.
1: c←C.
2: $y_{1}^{n},y_{2}^{n}\leftarrow S_{\gamma -1}^{k}$.
3: $w_{n}:={\mathrm{Ay}}_{1}^{n}+y_{2}^{n}$.
4: $z_{1}^{n}:=y_{1}^{n}+cs_{1}^{n}$ and $z_{2}^{n}:=y_{2}^{n}+cs_{2}^{n}$.
5: $com_{n}\leftarrow HomH\left(w_{n}\right)$, send out $h_{n}\leftarrow H_{3}\left(com_{n}\right)$.
6: Upon receiving $h_{i}$, search for $\left(com_{i},alert,bad_{7}\right)\leftarrow \mathrm{searchHash}\left(HT_{3},h_{i}\right)$.
7: **If** the flag $bad_{7}$ is set, then simulation fails with output (0,⊥). **If** the flag alert is set, then send out $com_{n}$.
8: $com:=com_{n}+com_{i}$.
9: Program random oracle $H_{0}$ to respond queries (m||com) with *c*. Set $HT_{0}[(m||com)]:=c$. If $HT_{0}\left[(m||com)\right]$ has been already set, set flag $bad_{8}$ and the simulation fails with output (0,⊥).
10: Send out $com_{n}$. Upon receiving $com_{i}$: - if $H_{3}\left(com_{i}\right)\neq h_{i}$: send out ABORT. - if the flag alert is set and $H_{3}\left(com_{i}\right)=h_{i}$: set the flag $bad_{9}$ and the simulation fails with output (0,⊥).
11: Otherwise, run rejection sampling, if it did not pass: send out RESTART and go to the step 1.
12: Otherwise, send out $\left(z_{1}^{n},z_{2}^{n}\right)$. Upon receiving RESTART, go to step 1.
13: Upon receiving $\left(z_{1}^{i},z_{2}^{i}\right)$, reconstruct $w_{i}:={\mathrm{Az}}_{1}^{i}+z_{2}^{i}-ct_{i}$ and check that $HomH\left(w_{i}\right)=com_{i}$, if not: send out ABORT.
14: Otherwise, set $z_{1}:=z_{1}^{n}+z_{1}^{i}$, $z_{2}:=z_{2}^{n}+z_{2}^{i}$ and output composed signature $\sigma :=(z_{1},z_{2},c)$.

Game 0 → Game 1:

The difference between Game 0 and Game 1 can be expressed using the bad events that can happen with the following probabilities:

- Pr[$bad_{7}$] is the probability that at least one collision occurs during at most $q_{h}+2q_{s}$ queries to the random oracle $H_{3}$ made by adversary or simulator. This means that two values $com_{j}\neq com_{j}^{\prime }$ were found such that $HT_{3}\left[com_{j}\right]=HT_{3}\left[com_{j}^{\prime }\right]$. As all of the responses of $H_{3}$ are chosen uniformly at random from ${\left\{0,1\right\}}^{l_{3}}$ and there are at most $q_{h}+2q_{s}$ queries to the random oracle $H_{3}$, the probability of at least one collision occurring can be expressed as $\frac{\left(\left(q_{h}+2q_{s}\right)\left(q_{h}+2q_{s}+1\right)\right)/2}{2^{l_{3}}}\leq \frac{{\left(q_{h}+2q_{s}+1\right)}^{2}}{2^{l_{3}+1}}$, where $l_{3}$ is the length of $H_{3}$ output.
- Pr[$bad_{8}$] is the probability that programming random oracle $H_{0}$ fails at least once during $q_{s}$ queries. This event can happen in the following two cases: $H_{3}\left(com_{n}\right)$ was previously queried by the adversary or it was not queried by the adversary:
  - Case 1: $H_{3}\left(com_{n}\right)$ has been already asked by adversary during at most $q_{h}+2q_{s}$ queries to $H_{3}$. This means that the adversary knows com and may have queried $H_{0}\left(m||com\right)$ before. This event corresponds to guessing the value of $com_{n}$.
    Let the uniform distribution over $Z_{p}^{b}$ be denoted as *X* and the distribution of HomH output be denoted as *Y*. As HomH is ϵ-regular (for some negligibly small ϵ), it holds that SD (X,Y)≤ϵ. Then, for any subset *T* of $Z_{p}^{b}$, by definition of statistical distance, it holds that Pr[X∈T]≤Pr[Y∈T]+ϵ. Therefore, for a uniform distribution *X*, the probability of guessing *Y* by *T* is bounded by $\frac{1}{|Z_{p}^{b}|}+\epsilon$.
    Since $com_{n}$ was produced by B in the beginning of the signing protocol completely independently from A, the probability that A queried $H_{3}\left(com_{n}\right)$ is at most $\frac{1}{|Z_{p}^{b}|}+\epsilon$ for each query.
  - Case 2: $HT_{0}\left[m||com\right]$ has been set by adversary or simulator by chance during at most $q_{h}+q_{s}$ prior queries to the $H_{0}$. Since A has not queried $H_{3}\left(com_{n}\right)$ before, adversary does not know $com_{n}$ and the view of A is completely independent from com. The probability that com occurred by chance in one of the previous queries to $H_{0}$ is at most $\left(q_{h}+q_{s}\right)\left(\frac{1}{|Z_{p}^{b}|}+\epsilon \right)$.
- Pr[$bad_{9}$] is the probability that the adversary predicted at least one of two outputs of the random oracle $H_{3}$ without making a query to it. In this case, there is no record in the hash table $HT_{3}$ that corresponds to the preimage $com_{j}$. This can happen with probability at most $\frac{2}{2^{l_{3}}}$ for each signing query.

Therefore, the difference between two games is

$$\begin{array}{c} |\mathrm{Pr}\left[G_{1}\right]-\mathrm{Pr}\left[G_{0}\right]|\leq \mathrm{Pr}\left[bad_{7}\right]+\mathrm{Pr}\left[bad_{8}\right]+\mathrm{Pr}\left[bad_{9}\right]\leq \\ \frac{{\left(q_{h}+2q_{s}+1\right)}^{2}}{2^{l_{3}+1}}+q_{s}\left(\left(q_{h}+2q_{s}\right)\left(\frac{1}{|Z_{p}^{b}|}+\epsilon \right)+\left(q_{h}+q_{s}\right)\left(\frac{1}{|Z_{p}^{b}|}+\epsilon \right)+\frac{2}{2^{l_{3}}}\right)=\\ \frac{{\left(q_{h}+2q_{s}+1\right)}^{2}}{2^{l_{3}+1}}+q_{s}\left(\left(\frac{1}{|Z_{p}^{b}|}+\epsilon \right)\cdot \left(2q_{h}+3q_{s}\right)+\frac{2}{2^{l_{3}}}\right). \end{array}$$

### Appendix A.3. Game 2

In Game 2, when the signature share gets rejected, simulator commits to a uniformly random vector $w_{n}$ from the ring $R_{q}$ instead of committing to a vector computed as ${\mathrm{Ay}}_{1}^{n}+y_{2}^{n}$. The simulator for the signing process in Game 2 is described in Algorithm 5.

Game 1 → Game 2:

The difference between Game 1 and Game 2 can be expressed with the probability that the adversary can distinguish simulated commitment with random $w_{n}$ from the real one when the rejection sampling algorithm does not pass. If the signature shares are rejected, it means that $z_{1}^{n}\geq \gamma -\beta$ or $z_{2}^{n}\geq \gamma -\beta$.

Let us assume that there exists an adversary D who succeeds in distinguish simulated commitment with random $w_{n}$ from the real one with nonnegligible probability:

$$\begin{array}{cc} \mathrm{Adv}\left(D\right)=\mathrm{Pr}[b=b^{\prime }:A\leftarrow R_{q}^{k\times k},s_{1},s_{2}\leftarrow S_{\eta -1}^{k}, & c\leftarrow C,y_{1},y_{2}\leftarrow S_{\gamma -1}^{k},\\ w_{0}\leftarrow {\mathrm{Ay}}_{1}+y_{2},w_{1}\leftarrow R_{q}^{k},b\leftarrow \left\{0,1\right\},h_{b}\leftarrow HomH & \left(w_{b}\right),b^{\prime }\leftarrow D\left(A,h_{b},c\right)\\ |y_{1}+cs_{1}\geq \gamma -\beta \;\mathrm{or}\;y_{2}+cs_{2} & \geq \gamma -\beta ]. \end{array}$$

Then, the adversary D can be used to construct an adversary $A_{R-MLWE}$ who solves the rejected Module-LWE for parameters (q,k,k,γ,U,β), where *U* is the uniform distribution. The adversary $A_{R-MLWE}$ is defined in Algorithm A6.

**Algorithm A5**$Sim_{Sign}\left(sk_{n},pk,m\right)$.
1: c←C.
2:$y_{1}^{n},y_{2}^{n}\leftarrow S_{\gamma -1}^{k}$.
3: $z_{1}^{n}:=y_{1}^{n}+cs_{1}^{n}$ and $z_{2}^{n}:=y_{2}^{n}+cs_{2}^{n}$.
4: Run rejection sampling; if it does not pass, proceed as follows:
1. $w_{n}\leftarrow R_{q}^{k}$.
2. $com_{n}\leftarrow HomH\left(w_{n}\right)$, send out $h_{n}\leftarrow H_{3}\left(com_{n}\right)$.
3. Upon receiving $h_{i}$, search for $\left(com_{i},alert,bad_{7}\right)\leftarrow \mathrm{searchHash}\left(HT_{3},h_{i}\right)$.
4. If the flag $bad_{7}$ is set, then simulation fails with output (0,⊥). If the flag alert is set, then send out $com_{n}$.
5. $com:=com_{n}+com_{i}$.
6. Program random oracle $H_{0}$ to respond queries (m||com) with *c*. Set $HT_{0}[(m||com)]:=c$. If $HT_{0}\left[(m||com)\right]$ has been already set, set flag $bad_{8}$ and the simulation fails with output (0,⊥).
7. Send out $com_{n}$. Upon receiving $com_{i}$: - if $H_{3}\left(com_{i}\right)\neq h_{i}$: send out ABORT. - if the flag alert is set and $H_{3}\left(com_{i}\right)=h_{i}$: set the flag $bad_{9}$ and the simulation fails with output (0,⊥).
8. Otherwise, send out RESTART and go to step 1.
5: If rejection sampling passes, proceed as follows:
1. $w_{n}:={\mathrm{Ay}}_{1}^{n}+y_{2}^{n}$.
2. $com_{n}\leftarrow HomH\left(w_{n}\right)$, send out $h_{n}\leftarrow H_{3}\left(com_{n}\right)$.
3. Upon receiving $h_{i}$, search for $\left(com_{i},alert,bad_{7}\right)\leftarrow \mathrm{searchHash}\left(HT_{3},h_{i}\right)$.
4. If the flag $bad_{7}$ is set, then simulation fails with output (0,⊥). If the flag alert is set, then continue.
5. $com:=com_{n}+com_{i}$.
6. Program random oracle $H_{0}$ to respond queries (m||com) with *c*. Set $HT_{0}[(m||com)]:=c$. If $HT_{0}\left[(m||com)\right]$ has been already set, set flag $bad_{8}$ and the simulation fails with output (0,⊥).
7. Send out $com_{n}$.Upon receiving $com_{i}$: - if $H_{3}\left(com_{i}\right)\neq h_{i}$: send out ABORT. - if the flag alert is set and $H_{3}\left(com_{i}\right)=h_{i}$: set the flag $bad_{9}$ and the simulation fails with output (0,⊥).
8. Otherwise, send out $\left(z_{1}^{n},z_{2}^{n}\right)$. Upon receiving RESTART, go to step 1.
9. Upon receiving $\left(z_{1}^{i},z_{2}^{i}\right)$, reconstruct $w_{i}:={\mathrm{Az}}_{1}^{i}+z_{2}^{i}-ct_{i}$ and check that $HomH\left(w_{i}\right)=com_{i}$, if not: send out ABORT.
10. Otherwise, set $z_{1}:=z_{1}^{n}+z_{1}^{i}$, $z_{2}:=z_{2}^{n}+z_{2}^{i}$ and output composed signature $\sigma :=(z_{1},z_{2},c)$.

**Algorithm A6**$A_{R-MLWE}\left(A,w_{b},c\right)$.
1: $h_{b}\leftarrow HomH\left(w_{b}\right)$
2: $b^{\prime }\leftarrow D\left(A,h_{b},c\right)$
3: **return** $b^{\prime }$

As a consequence, the difference between the two games is bounded by the following:

$$|\mathrm{Pr}\left[G_{2}\right]-\mathrm{Pr}\left[G_{1}\right]|\leq q_{s}\cdot {\mathrm{Adv}}_{\left(q,k,k,\gamma ,U,\beta \right)}^{R-\mathrm{MLWE}}$$

### Appendix A.4. Game 3

In Game 3, the simulator does not generate the signature shares honestly and thus does not perform rejection sampling honestly. Rejection sampling is simulated as follows:

- **Rejection case**: with probability $1-{\left(1-\frac{|S_{\gamma -\beta -1}^{k}|}{|S_{\gamma -1}^{k}|}\right)}^{2}$ simulator generates commitment to the random $w_{n}$ as in the previous game.
- **Otherwise**, sample signature shares from the set $S_{\gamma -\beta -1}$ and compute $w_{n}$ out of it.

The simulator for the signing process in Game 3 is described in Algorithm A7.

Game 2 → Game 3:

The signature shares generated in Algorithm A7 are indistinguishable from the real ones because of the $\epsilon_{ZK}$-naHVZK property of the underlying identification scheme from [13], appendix B. Therefore, the difference between Game 2 and Game 3 can be defined as follows:

$$|\mathrm{Pr}\left[G_{3}\right]-\mathrm{Pr}\left[G_{2}\right]|\leq \epsilon_{ZK}$$

According to the proof from [13], $\epsilon_{ZK}=0$ for the underlying identification scheme.

**Algorithm A7**$Sim_{Sign}\left(t_{n},pk,m\right)$.
1: With probability $1-{\left(1-\frac{|S_{\gamma -\beta -1}^{k}|}{|S_{\gamma -1}^{k}|}\right)}^{2}$, proceed as follows: 1. c←C. 2. $w_{n}\leftarrow R_{q}^{k}$. 3. $com_{n}\leftarrow HomH\left(w_{n}\right)$, send out $h_{n}\leftarrow H_{3}\left(com_{n}\right)$. 4. Upon receiving $h_{i}$, search for $\left(com_{i},alert,bad_{7}\right)\leftarrow \mathrm{searchHash}\left(HT_{3},h_{i}\right)$. 5. If the flag $bad_{7}$ is set, then simulation fails with output (0,⊥). If the flag alert is set, then send out $com_{n}$. 6. $com:=com_{n}+com_{i}$. 7. Program random oracle $H_{0}$ to respond queries (m||com) with *c*. Set $HT_{0}[(m||com)]:=c$. If $HT_{0}\left[(m||com)\right]$ has been already set, set flag $bad_{8}$ and the simulation fails with output (0,⊥). 8. Send out $com_{n}$. Upon receiving $com_{i}$: - if $H_{3}\left(com_{i}\right)\neq h_{i}$: send out ABORT. - if the flag alert is set and $H_{3}\left(com_{i}\right)=h_{i}$: set the flag $bad_{9}$ and the simulation fails with output (0,⊥). 9. Otherwise, send out RESTART and go to step 1.
2: Otherwise, proceed as follows: 1. c←C. 2. $z_{1}^{n}\leftarrow S_{\gamma -\beta -1}^{k}$ and $z_{2}^{n}\leftarrow S_{\gamma -\beta -1}^{k}$. 3. $w_{n}:={\mathrm{Az}}_{1}^{n}+z_{2}^{n}-ct_{n}$. 4. $com_{n}\leftarrow HomH\left(w_{n}\right)$, send out $h_{n}\leftarrow H_{3}\left(com_{n}\right)$. 5. Upon receiving $h_{i}$, search for $\left(com_{i},alert,bad_{7}\right)\leftarrow \mathrm{searchHash}\left(HT_{3},h_{i}\right)$. 6. If the flag $bad_{7}$ is set, then simulation fails with output (0,⊥). If the flag alert is set, then continue. 7. $com:=com_{n}+com_{i}$. 8. Program random oracle $H_{0}$ to respond queries (m||com) with *c*. Set $HT_{0}[(m||com)]:=c$. If $HT_{0}\left[(m||com)\right]$ has been already set, set flag $bad_{8}$ and the simulation fails with output (0,⊥). 9. Send out $com_{n}$. Upon receiving $com_{i}$: - if $H_{3}\left(com_{i}\right)\neq h_{i}$: send out ABORT. - if the flag alert is set and $H_{3}\left(com_{i}\right)=h_{i}$: set the flag $bad_{9}$ and the simulation fails with output (0,⊥). 10. Otherwise, send out $\left(z_{1}^{n},z_{2}^{n}\right)$. Upon receiving RESTART, go to step 1. 11. Upon receiving $\left(z_{1}^{i},z_{2}^{i}\right)$, reconstruct $w_{i}:={\mathrm{Az}}_{1}^{i}+z_{2}^{i}-ct_{i}$ and check that $HomH\left(w_{i}\right)=com_{i}$, if not: send out ABORT. 12. Otherwise, set $z_{1}:=z_{1}^{n}+z_{1}^{i}$, $z_{2}:=z_{2}^{n}+z_{2}^{i}$ and output composed signature $\sigma :=(z_{1},z_{2},c)$.

### Appendix A.5. Game 4

Now, the signing process does not rely on the actual secret key share of the honest party $P_{n}$. In the next games, the key generation process is changed so that it does not use secret keys as well. In this game, the simulator is given a predefined uniformly random matrix $A\leftarrow R_{q}^{k\times k}$, and the simulator defines its own matrix share out of it. By definition, the algorithm B (Algorithm A1) receives a pre-generated public key pk as the input. Therefore, the simulator in Game 4 is given a predefined matrix A, and in the later games, the simulator is changed so that it receives the entire public key and uses it to compute its shares $A_{n},t_{n}$. The simulator for the key generation process in Game 4 is described in Algorithm A8.

**Algorithm A8**$Sim_{KeyGen}(par,$A).
1: Send out $hk_{n}\leftarrow {\left\{0,1\right\}}^{l_{1}}$
2: Upon receiving $hk_{i}$:
- search for $\left(A_{i},alert,bad_{1}\right)\leftarrow \mathrm{searchHash}\left(HT_{1},hk_{i}\right)$. - if the flag $bad_{1}$ is set, then simulation fails with output (0,⊥). - if the flag alert is set, then sample $A_{n}\leftarrow R_{q}^{k\times k}$. Otherwise, define $A_{n}:=A-A_{i}$.
3: Program random oracle $H_{1}$ to respond queries $A_{n}$ with $hk_{n}$. Set $HT_{1}\left[A_{n}\right]:=hk_{n}$. If $HT_{1}\left[A_{n}\right]$ has been already set, then set the flag $bad_{2}$ and the simulation fails with output (0,⊥).
4: Send out $A_{n}$. Upon receiving $A_{i}$: - if $H_{1}\left(A_{i}\right)\neq hk_{i}$: send out ABORT. - if the flag alert is set and $H_{1}\left(A_{i}\right)=hk_{i}$: set the flag $bad_{3}$ and the simulation fails with output (0,⊥).
5: ($s_{1}^{n}$, $s_{2}^{n}$) $\leftarrow S_{\eta }^{k}\times S_{\eta }^{k}$.
6: $t_{n}:={\mathrm{As}}_{1}^{n}+s_{2}^{n}$, send out $comk_{n}:=H_{2}\left(t_{n}\right)$.
7: Upon receiving $comk_{i}$, send out $t_{n}$.
8: Upon receiving $t_{i}$, check that $H_{2}\left(t_{i}\right)=comk_{i}$. If not: send out ABORT.
9: Otherwise, $t:=t_{n}+t_{i}$, pk:=(A,t) and $sk:=(A,t_{i},s_{n},s_{n}^{\prime })$.

Game 3 → Game 4:

The distribution of public matrix A does not change between Game 3 and Game 4. The difference between Game 3 and Game 4 can be expressed using bad events that happen with the following probabilities:

- Pr[$bad_{1}$] is the probability that at least one collision occurs during at most $q_{h}$ queries to the random oracle $H_{1}$ made by adversary or simulator. This can happen with probability at most $\frac{q_{h}\left(q_{h}+1\right)/2}{2^{l_{1}+1}}$, where $l_{1}$ is the length of $H_{1}$ output.
- Pr[$bad_{2}$] is the probability that programming random oracle $H_{1}$ fails, which happens if $H_{1}\left(A_{n}\right)$ has been previously asked by adversary during at most $q_{h}$ queries to the random oracle $H_{1}$. This event corresponds to guessing random $A_{n}$, for each query the probability of this event is bounded by $\frac{1}{q^{n\cdot k\cdot k}}$.
- Pr[$bad_{3}$] is the probability that adversary predicted at least one of two outputs of the random oracle $H_{1}$ without making a query to it. This can happen with probability at most $\frac{2}{2^{l_{1}}}$.

Therefore, the difference between the two games is

$$|\mathrm{Pr}\left[G_{4}\right]-\mathrm{Pr}\left[G_{3}\right]|\leq \mathrm{Pr}\left[bad_{1}\right]+\mathrm{Pr}\left[bad_{2}\right]+\mathrm{Pr}\left[bad_{3}\right]\leq \frac{\left(q_{h}+1\right)q_{h}}{2^{l_{1}+1}}+\frac{q_{h}}{q^{n\cdot k\cdot k}}+\frac{2}{2^{l_{1}}}$$

### Appendix A.6. Game 5

In Game 5, the simulator picks public key share $t_{n}$ randomly from the ring instead of computing it using secret keys. The simulator for the key generation process in Game 5 is described in Algorithm A9.

**Algorithm A9**$Sim_{KeyGen}\left(par,A\right)$.
1: Send out $hk_{n}\leftarrow {\left\{0,1\right\}}^{l_{1}}$.
2: Upon receiving $hk_{i}$: - search for $\left(A_{i},alert,bad_{1}\right)\leftarrow \mathrm{searchHash}\left(HT_{1},hk_{i}\right)$. - if the flag $bad_{1}$ is set, then simulation fails with output (0,⊥). - if the flag alert is set, then sample $A_{n}\leftarrow R_{q}^{k\times k}$. Otherwise, define $A_{n}:=A-A_{i}$.
3: Program random oracle $H_{1}$ to respond queries $A_{n}$ with $hk_{n}$. Set $HT_{1}\left[A_{n}\right]:=hk_{n}$. If $HT_{1}\left[A_{n}\right]$ has been already set, then set the flag $bad_{2}$ and the simulation fails with output (0,⊥).
4: Send out $A_{n}$. Upon receiving $A_{i}$: - if $H_{1}\left(A_{i}\right)\neq hk_{i}$: send out ABORT. - if the flag alert is set and $H_{1}\left(A_{i}\right)=hk_{i}$: set the flag $bad_{3}$ and the simulation fails with output (0,⊥).
5: $t_{n}\leftarrow R_{q}^{k}$, send out $comk_{n}=H_{2}\left(t_{n}\right)$.
6: Upon receiving $comk_{i}$, send out $t_{n}$.
7: Upon receiving $t_{i}$, check that $H_{2}\left(t_{i}\right)=comk_{i}$. If not: send out ABORT.
8: Otherwise, $t:=t_{n}+t_{i}$, pk:=(A,t).

Game 4 → Game 5:

In Game 5, public key share $t_{n}$ is sampled uniformly at random from $R_{q}^{k}$ instead of computing it as ${\mathrm{As}}_{1}^{n}+s_{2}^{n}$, where $s_{1}^{n},s_{2}^{n}$ are random elements from $S_{\eta }^{k}$. As matrix A follows the uniform distribution over $R_{q}^{k\times k}$, if adversary can distinguish between Game 3 and Game 4, this adversary can be used as a distinguisher that breaks the decisional Module-LWE problem for parameters (q,k,k,η,U), where *U* is the uniform distribution.

Therefore, the difference between two games is bounded by the advantage of adversary in breaking decisional Module-LWE:

$$|\mathrm{Pr}\left[G_{5}\right]-\mathrm{Pr}\left[G_{4}\right]|\leq {\mathrm{Adv}}_{\left(q,k,k,\eta ,U\right)}^{\mathrm{Dec}-\mathrm{MLWE}}$$

### Appendix A.7. Game 6

In Game 6, the simulator uses as input a random resulting public key $t\in R_{q}^{k}$ to compute its own share $t_{n}$. The simulator for the key generation process in Game 6 is described in Algorithm A10.

Game 5 → Game 6:

The distributions of $t,t_{n}$ do not change with respect to Game 5. The difference between Game 5 and Game 6 can be expressed using bad events that happen with the following probabilities:

- Pr[$bad_{4}$] is the probability that at least one collision occurs during at most $q_{h}$ queries to the random oracle $H_{2}$ made by adversary or simulator. This can happen with probability at most $\frac{q_{h}\left(q_{h}+1\right)/2}{2^{l_{2}+1}}$, where $l_{2}$ is the length of $H_{2}$ output.
- Pr[$bad_{5}$] is the probability that programming random oracle $H_{2}$ fails, which happens if $H_{2}\left(t_{n}\right)$ was previously asked by adversary during at most $q_{h}$ queries to the random oracle $H_{2}$. This event corresponds to guessing a uniformly random $t_{n}\in R_{q}^{k}$, for each query the probability of this event is bounded by $\frac{1}{q^{n\cdot k}}$.
- Pr[$bad_{6}$] is the probability that adversary predicted at least one of two outputs of the random oracle $H_{2}$ without making a query to it. This can happen with probability at most $\frac{2}{2^{l_{2}}}$.

Therefore, the difference between the two games is

$$|\mathrm{Pr}\left[G_{6}\right]-\mathrm{Pr}\left[G_{5}\right]|\leq \mathrm{Pr}\left[bad_{4}\right]+\mathrm{Pr}\left[bad_{5}\right]+\mathrm{Pr}\left[bad_{6}\right]\leq \frac{\left(q_{h}+1\right)q_{h}}{2^{l_{2}+1}}+\frac{q_{h}}{q^{n\cdot k}}+\frac{2}{2^{l_{2}}}$$

**Algorithm A10**$Sim_{KeyGen}\left(par,A,t\right)$.
1: Send out $hk_{n}\leftarrow {\left\{0,1\right\}}^{l_{1}}$.
2: Upon receiving $hk_{i}$: - search for $\left(A_{i},alert,bad_{1}\right)\leftarrow \mathrm{searchHash}\left(HT_{1},hk_{i}\right)$. - if the flag $bad_{1}$ is set, then simulation fails with output (0,⊥). - if the flag alert is set, then sample $A_{n}\leftarrow R_{q}^{k\times k}$. Otherwise, define $A_{n}:=A-A_{i}$.
3: Program random oracle $H_{1}$ to respond queries $A_{n}$ with $hk_{n}$. Set $HT_{1}\left[A_{n}\right]:=hk_{n}$. If $HT_{1}\left[A_{n}\right]$ has been already set, then set the flag $bad_{2}$ and the simulation fails with output (0,⊥).
4: Send out $A_{n}$. Upon receiving $A_{i}$: - if $H_{1}\left(A_{i}\right)\neq hk_{i}$: send out ABORT. - if the flag alert is set and $H_{1}\left(A_{i}\right)=hk_{i}$: set the flag $bad_{3}$ and the simulation fails with output (0,⊥).
5: Send out $comk_{n}\leftarrow {\left\{0,1\right\}}^{l_{2}}$.
6: Upon receiving $comk_{i}$, search for $\left(t_{i},alert,bad_{4}\right)\leftarrow \mathrm{searchHash}\left(HT_{2},comk_{i}\right)$.
7: If the flag $bad_{4}$ is set, then simulation fails with output (0,⊥).
8: Compute public key share: - If the flag alert is set, $t_{n}\leftarrow R_{q}^{k}$. - Otherwise, $t_{n}:=t-t_{i}$.
9: Program random oracle $H_{2}$ to respond queries $t_{n}$ with $comk_{n}$. Set $HT_{2}\left[t_{n}\right]:=comk_{n}$. If $HT_{2}\left[t_{n}\right]$ has been already set, set flag $bad_{5}$ and the simulation fails with output (0,⊥).
10: Send out $t_{n}$. Upon receiving $t_{i}$: - if $H_{2}\left(t_{i}\right)\neq comk_{i}$: send out ABORT. - if the flag alert is set and $H_{2}\left(t_{i}\right)=comk_{i}$: set the flag $bad_{6}$ and simulation fails with output (0,⊥).
11: Otherwise, $t:=t_{n}+t_{i}$, pk:=(A,t).

### Appendix A.8. Forking Lemma

Now, both key generation and signing do not rely on the actual secret key share of the honest party $P_{n}$. In order to conclude the proof, it is needed to invoke forking lemma to receive two valid forgeries that are constructed using the same commitment $com=com^{\prime }$ but different challenges $c\neq c^{\prime }$.

Currently, the combined public key consists of matrix A uniformly distributed in $R_{q}^{k\times k}$ and vector t uniformly distributed in $R_{q}^{k}$. We want to replace it with Module-SIS instance $[\begin{array}{c} A^{\prime }|I \end{array}]$, where $A^{\prime }\in R_{q}^{k\times (k+1)}$. The view of adversary will not be changed if we set $A^{\prime }= [\begin{array}{c} A|t \end{array}]$.

Let us define an input generation algorithm IG such that it produces the following input: (A,t) for the $F_{B}$. Now, let us construct $B^{\prime }$ around the previously defined simulator B. $B^{\prime }$ invokes the forking algorithm $F_{B}$ on the input (A,t).

As a result, with probability frk two valid forgeries $out=(com,c,z_{1},z_{2},m)$ and $out^{\prime }=\left(com^{\prime },c^{\prime },z_{1}^{\prime },z_{2}^{\prime },m^{\prime }\right)$ are obtained. Here, by the construction of $F_{B}$, it holds that $c\neq c^{\prime }$, $com=com^{\prime },m=m^{\prime }$. The probability frk satisfiesollowing:

$$\mathrm{Pr}\left[G_{6}\right]=acc\leq \frac{q_{h}+q_{s}+1}{\left|C\right|}+\sqrt{(q_{h}+q_{s}+1)\cdot frk}$$

Since both signatures are valid, it holds that

$$HomH\left({\mathrm{Az}}_{1}+z_{2}-ct\right)=com=com^{\prime }=HomH\left({\mathrm{Az}}_{1}^{\prime }+z_{2}^{\prime }-c^{\prime }t\right)$$

Let us examine the following cases:

**Case 1** **:** ${\mathrm{Az}}_{1}+z_{2}-ct\neq {\mathrm{Az}}_{1}^{\prime }+z_{2}^{\prime }-c^{\prime }t$, and $B^{\prime }$ is able to break the collision resistance of the hash function (that is hard under the worst-case difficulty of finding short vectors in cyclic/ideal lattices), as was proven in [35,36].

**Case 2** **:** ${\mathrm{Az}}_{1}+z_{2}-ct={\mathrm{Az}}_{1}^{\prime }+z_{2}^{\prime }-c^{\prime }t$. It can be rearranged as ${\mathrm{Az}}_{1}-{\mathrm{Az}}_{1}^{\prime }+z_{2}-z_{2}-ct+c^{\prime }t=0$, and this, in turn, leads to

$$[\begin{array}{c} A|I|t \end{array}] [\begin{array}{c} z_{1}-z_{1}^{\prime }\\ z_{2}-z_{2}^{\prime }\\ c^{\prime }-c \end{array}]=0$$

Now, recall that $[\begin{array}{c} A|I|t \end{array}]$ is an instance of Module-SIS problem; this means that we found a solution for Module-SIS with parameters (q,k,k+1,ξ), where $\xi \leq 2(\gamma_{2}-\beta_{2})$. Therefore, the probability frk is the following:

$$frk\leq {\mathrm{Adv}}_{\left(q,k,k+1,\xi \right)}^{\mathrm{MSIS}}+{\mathrm{Adv}}^{\mathrm{CR}}$$

Finally, taking into account that the underlying identification scheme has perfect naHVZK (i.e., $\epsilon_{ZK}=0$), the advantage of the adversary is bounded by the following:

$$\begin{array}{cc} {\mathrm{Adv}}^{\mathrm{DS}-\mathrm{UF}-\mathrm{CMA}}\left(A\right)\leq \frac{{\left(q_{h}+2q_{s}+1\right)}^{2}}{2^{l_{3}+1}} & +q_{s}\cdot \left(\left(\frac{1}{|Z_{p}^{b}|}\right)\cdot \left(2q_{h}+3q_{s}\right)+\frac{2}{2^{l_{3}}}\right)+\\ q_{s}\cdot {\mathrm{Adv}}_{\left(q,k,k,\gamma ,U,\beta \right)}^{R-\mathrm{MLWE}}+\frac{\left(q_{h}+1\right)q_{h}}{2^{l_{1}+1}} & +\frac{q_{h}}{q^{n\cdot k\cdot k}}+\frac{2}{2^{l_{1}}}+{\mathrm{Adv}}_{\left(q,k,k,\eta ,U\right)}^{\mathrm{Dec}-\mathrm{MLWE}}+\\ \frac{\left(q_{h}+1\right)q_{h}}{2^{l_{2}+1}}+\frac{q_{h}}{q^{n\cdot k}}+\frac{2}{2^{l_{2}}}+\frac{q_{h}+q_{s}+1}{\left|C\right|} & +\sqrt{\left(q_{h}+q_{s}+1\right)\cdot \left({\mathrm{Adv}}_{\left(q,k,k+1,\xi \right)}^{\mathrm{MSIS}}+{\mathrm{Adv}}^{\mathrm{CR}}\right)} \end{array}$$

☐
